# Effect of perioperative acupuncture-assisted general anesthesia on the anesthetic dosage required in adult surgical patients: a network meta-analysis of randomized controlled trials

**DOI:** 10.3389/fmed.2023.1133585

**Published:** 2023-05-10

**Authors:** Meihua Qiu, Chuanxiong Li, Tong Sun, Qianwen Ruan

**Affiliations:** ^1^Department of Rehabilitation Medicine, The Affiliated Hospital of Yunnan University, Kunming, Yunnan, China; ^2^The Second Affiliated Hospital of Kunming Medical University, Kunming, Yunnan, China

**Keywords:** electroacupuncture, general anesthesia, manual acupuncture, network meta-analysis, TEAS

## Abstract

**Objective:**

To determine the comparative effects of acupuncture and related techniques-assisted general anesthesia (GA) on the total dosage of main anesthetic drugs administered during surgery.

**Methods:**

The following data bases were searched on June 30, 2022: Embase, Cochrane, PubMed, Web of Science, CBM, CNKI, WANFANG and VIP to find randomized controlled trials (RCTs). A random-effects Bayesian network meta-analysis and subgroup analysis were employed. The GRADE system was applied to make evidence quality assessments. The intraoperative total doses of propofol and remifentanil were the primary and secondary outcomes, respectively. The weighted mean difference (WMD) with 95% confidence intervals (CI) were determined to measure the size of any potential effect.

**Results:**

Seventy-six RCTs that involved 5,877 patients were included in the analysis. Compared with GA, a significant decrease in the total dose of propofol was found for manual acupuncture (MA) assisted GA (WMD = −101.26 mg, 95% CI [−172.98, −27.06]) with moderate quality, electroacupuncture (EA) assisted GA (WMD = −54.25 mg, 95% CI [−87.25, −22.37]) with moderate quality and transcutaneous electrical acupoint stimulation (TEAS) assisted GA (WMD = −39.99 mg, 95% CI [−57.96, −22.73]) with moderate quality, respectively. A significant reduction in the total dose of remifentanil was found in favor of EA-assisted GA (WMD = −372.33 μg, 95% CI [−558.44, −196.43]) with low quality and TEAS-assisted GA (WMD = −215.77 μg, 95% CI [−305.23, −128.04]) with low quality. According to the surface under cumulative ranking area (SUCRA), MA-assisted GA and EA-assisted GA ranked first in the reduction of the total dosage of propofol and remifentanil administered, with a probability of 0.85 and 0.87, respectively.

**Conclusions:**

Both EA- and TEAS-assisted GA significantly reduced the intraoperative total dosage of propofol and remifentanil administered. EA produced the greatest reduction in these two outcomes compared to TEAS. Although all the comparisons are low to moderate based on GRADE evidence, EA seems to be an advisable acupuncture technique to reduce the dosage of anesthetic drugs required in surgical patients under GA.

## 1. Introduction

Propofol is an effective intravenous hypnotic drug, which is widely used for anesthesia induction and maintenance of general anesthesia (GA) and sedation during surgery. However, the most common side effect of propofol administration is dose-dependent hypotension (1). Propofol also affects neuronal regeneration, learning and memory (2). Remifentanil, an opioid, is often administered intraoperatively due to its very rapid pharmacokinetic properties, but the use of high-dose remifentanil is associated with a higher risk of hyperalgesia and tolerance (3, 4). Therefore, decreasing the dosage of anesthetic drugs during surgery will not only reduce medical expenses, but also the side effects of anesthetic drugs, which is conducive to the recovery of patients.

In the 1950's, China began to put forward the application of acupuncture anesthesia for surgery and in the 1960's, it was established as an effective anesthesia method (5). With the continuous development and exploration of acupuncture anesthesia techniques, the application of this techniques has shifted from a simple intraoperative to multiple perioperative stages. Current research has revealed that perioperative acupuncture has the potential to become an active intervention under the concept of ERAS (Enhanced Recovery After Surgery) (6).

At present, acupuncture anesthesia is commonly used in combination with anesthetics, a technique termed acupuncture-assisted anesthesia (AAA) or combined acupuncture-medicine anesthesia (CAMA). In 2015, a meta-analysis on acupuncture-assisted GA (7), which included 12 randomized controlled trials (RCTs), quantitatively showed that acupuncture-assisted GA could reduce the total dosage of propofol administered during surgery compared with conventional GA. Since 2018, a large number of RCTs of acupuncture-assisted GA have been published. In 2021, a literature review qualitatively summarized the effect of AAA in lowering the anesthesia dosage during thyroid, abdominal and anorectal surgery (8). Here we provide an updated meta-analysis on this topic. In addition, there is a paucity of direct comparative evidence for any differences in the effects of various acupuncture techniques on the reduction of intraoperative anesthetic drug doses. Which acupuncture technique should be chosen clinically to assist GA still confounds clinicians and policy makers.

Therefore, our goal was to retrieve and analyze all RCTs reporting on acupuncture-assisted GA, and to use the Bayesian network meta-analysis method to investigate the comparative effects of different acupuncture-assisted GA techniques on the total dosage of the commonly used anesthetic drugs, propofol and remifentanil, administered during surgery. The aim was to provide evidence-based reasons for the clinical selection of the best treatment scheme for acupuncture-assisted GA from the perspective of lowering the normally required dosage of anesthetics.

## 2. Methods

The study strictly followed the PRISMA extension for network meta-analysis guidelines.

### 2.1. Study inclusion criteria

Type of study: RCTs in Chinese and English. Study subjects: adult patients (≥ 18 years) who completed the surgery under GA, regardless of age, gender, race, type of surgery, method of GA, course of disease or the source of cases. A standard anesthetic protocol was followed and the surgeries were performed by the same surgical team. Interventions included the implementation of a certain acupuncture technique (such as electroacupuncture (EA), manual acupuncture (MA), transcutaneous electrical acupoint stimulation (TEAS), auricular acupuncture (AA), acupressure (AP) together with conventional GA. The time of the acupuncture intervention was defined as the period from 1 to 3 days before surgery, 30 min before the induction of GA, to the completion of each operation. The control group received conventional GA without acupuncture treatment (including sham acupuncture + GA and pure GA, namely sham and blank controls). The methods of GA included total intravenous anesthesia (TIVA) and intravenous/inhalation combined anesthesia (CIIA). The types of anesthetics used during GA were required to be the same in the intervention and control groups. The primary outcome was the total dosage of the main sedative intravenous anesthetic propofol and the secondary outcome was the total amount of the main analgesic intravenous anesthetic, remifentanil administered during surgery.

The exclusion criteria were: patients with GA combined with local anesthesia; studies involving the combination of two or more acupuncture techniques; the full text was not available; no response after contacting the authors; no data on the outcomes of interest or basic subject information; or a duplicate publication of the literature.

### 2.2. Source of literature and search

Two researchers (QWR and CXL) independently carried out a literature search of four English electronic databases (the Cochrane Library, PubMed, Embase and Web of Science) and four Chinese databases [China Biology Medicine (CBM), China National Knowledge Infrastructure (CNKI), WANFANG and China Science and Technology Journal (VIP)]. Disagreements or discrepancies were resolved by consultation with a third reviewer (MHQ). The retrieval time limit was from the establishment of the database to June 30, 2022. Article retrieval mainly consisted of three components, namely acupuncture, GA and RCT. The languages were restricted to English and Chinese. See Appendix 1 for the specific retrieval strategy for each database.

### 2.3. Screening of literature and the extraction of data

The literature search results were imported into EndNote (Thomson Reuters, Carlsbad, CA, USA). Two researchers (MHQ and TS) conducted independent screening of the literature, data extraction and cross-checks. If there was a disagreement, the parties discussed each article to reach consensus, else a third investigator assisted in the adjudication process (*vide supra*). Excel software was used to create a spreadsheet to store the data extracted from the included studies. The extracted contents included: (1) Basic information, study title, first author's name, publication year; (2) Baseline characteristics, sample size of the intervention and control group, gender, age and weight of patients, surgery type and duration and the anesthesia method; (3) Intervention, type of acupuncture technique, selected acupoints, frequency and intensity of the electronic instruments used, administration timing and duration of acupuncture; (4) Supporting information for risk of bias assessment; randomization method; allocation concealment; blinding and data integrity; and (5) Outcome, mean and standard deviation of total dosage of propofol and remifentanil.

### 2.4. Assessment of evidence quality

On the premise of hiding the names of the study authors, four researchers (QWR, CXL, MHQ, TS) assessed the risk of bias of the included studies using the Cochrane Risk-of-Bias tool 2.0 (9), and was used to draw the summary figure. Because the outcomes of interest (total dosages of propofol and remifentanil) are objective indicators, the detection bias of the two outcomes was the same, thus the risk of bias assessment was only conducted once. The quality of evidence contributing to each network was evaluated by the Grading of Recommendations Assessment, Development and Evaluation (GRADE), to determine the evidence quality of the two outcomes according to study limitations, imprecise data, inconsistency, indirectness and potential publication bias (10). If there were differences of opinions during the evaluation process and no agreement could be reached after discussion, the third party's view was adopted for the adjudication process.

### 2.5. Synthesis and analysis of data

#### 2.5.1. Methods for direct comparisons

A normal pairwise meta-analysis was conducted using a random effects model. The weighted mean difference (WMD) and 95% confidence interval (CI) of each outcome were determined to measure the potential size of any effect. The *I*^2^ statistic was used to calculate heterogeneity as a measure of the proportion of overall variation caused by heterogeneity between studies.

#### 2.5.2. Methods to make indirect or mixed comparisons

The Markov Chain Monte Carlo technique was employed for Bayesian inference (11) and random and fixed effects models were fitted. The degree of fit to the models was judged according to deviance information criterion (DIC), and the model with the smallest DIC value was adopted. League plots were drawn based on effect values for pairwise comparisons between interventions. The optimal probability values and the surface under cumulative ranking area (SUCRA) of all the interventions were calculated to show the likelihood of each intervention being the best intervention, so as to rank each intervention in terms of efficacy. The closer the SUCRA value to 1, the better the efficacy of the intervention.

#### 2.5.3. Assessment of consistency, heterogeneity and transitivity

When a closed loop was formed in the network evidence graph, the inconsistency factor (IF) was calculated to evaluate whether there was local inconsistency in each closed loop. When the IF value was close to 0 or the 95% CI contained a 0, it indicated that the results of direct and indirect comparisons were consistent or inconsistent. At the same time, the node splitting method was used to assess model inconsistency. If the corresponding *P*-values of each split node comparison were > 0.05, this indicated that the difference between direct and indirect comparisons was not significant.

Global heterogeneity was determined using the *I*^2^ statistic, a prediction interval value that tests the heterogeneity between studies. The uncertainty of the heterogeneity effect was defined as inconsistency between the CIs of the relative treatment effect and its prediction interval.

The transitivity hypothesis of the analysis was assessed by comparing the distribution of clinical variables that can be used as effect modification factors for comparisons of treatments.

#### 2.5.4. Subgroup analysis and publication bias

Contribution plots were constructed to evaluate the contribution of each direct comparison to the estimated summary effects of each network meta-analysis (12). In order to establish if the results were affected by the study characteristics, a network meta-analysis of the subgroups was performed according to age, GA method, comparison with sham acupuncture + GA or GA, number of acupoints, administration timing of the acupuncture, surgery type and duration. In addition, adjusted comparison funnel plots were drawn to identify small-sample effects of intervention networks and publication bias. Since some of the included trials were not specifically designed to assess the effect of acupuncture-assisted GA on the anesthesia dosage, sensitivity analysis of network meta-analysis was narrowed to trials specifically designed to assess the effect of acupuncture-assisted GA on the anesthesia dosage to validate the robustness of the results.

All the statistical analyses were carried out using R 4.2.0 (Network meta-analysis (GEMTC package), global heterogeneity test, transitivity and SUCRA graphs), and STATA ver. 13.0 for pairwise meta-analysis, tests for inconsistency, local heterogeneity and funnel plots.

## 3. Results

### 3.1. Included articles characteristics

**Figure 1:**
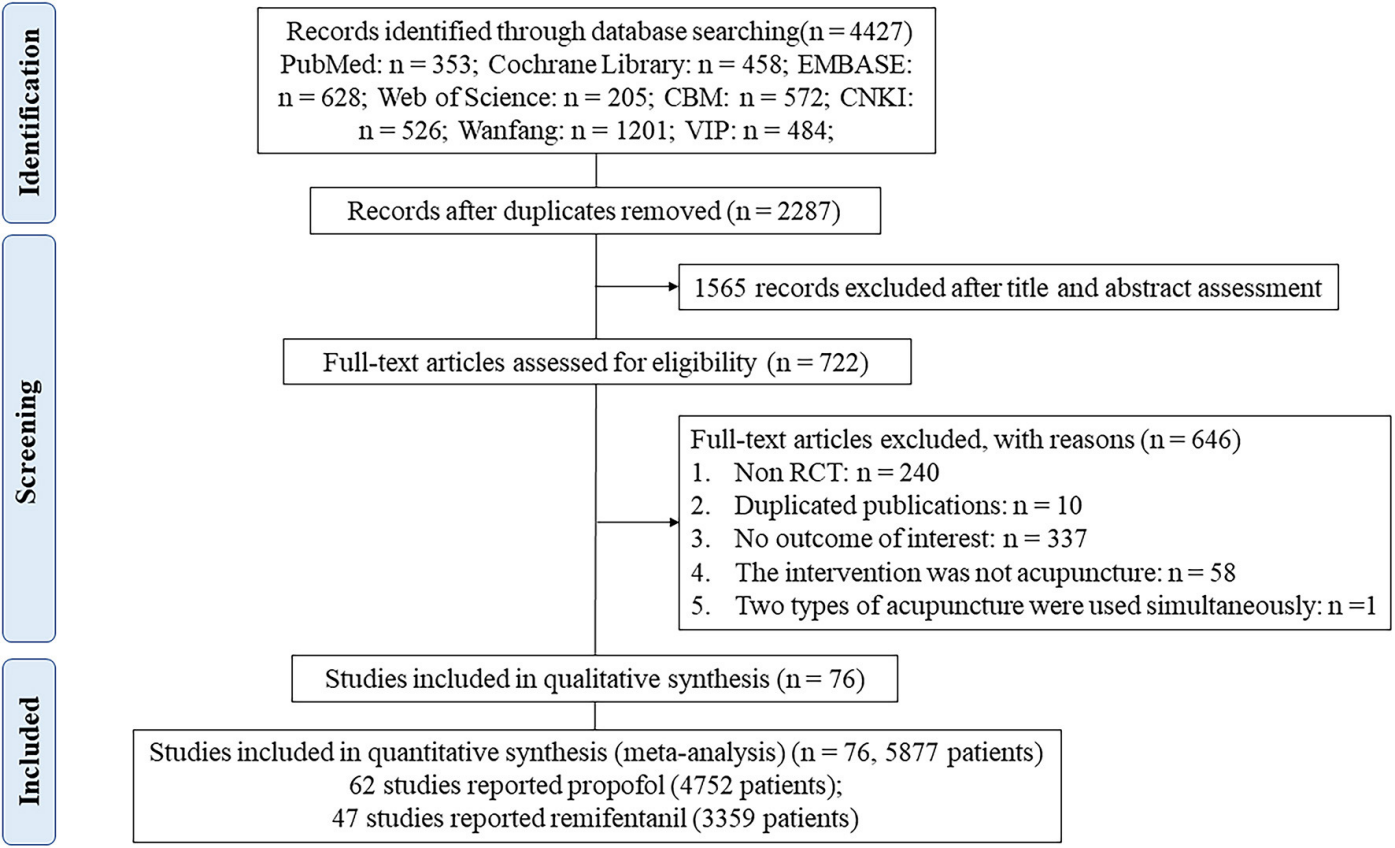
Flowchart of literature screening on acupuncture-assisted general anesthesia.

**Figure 2:**
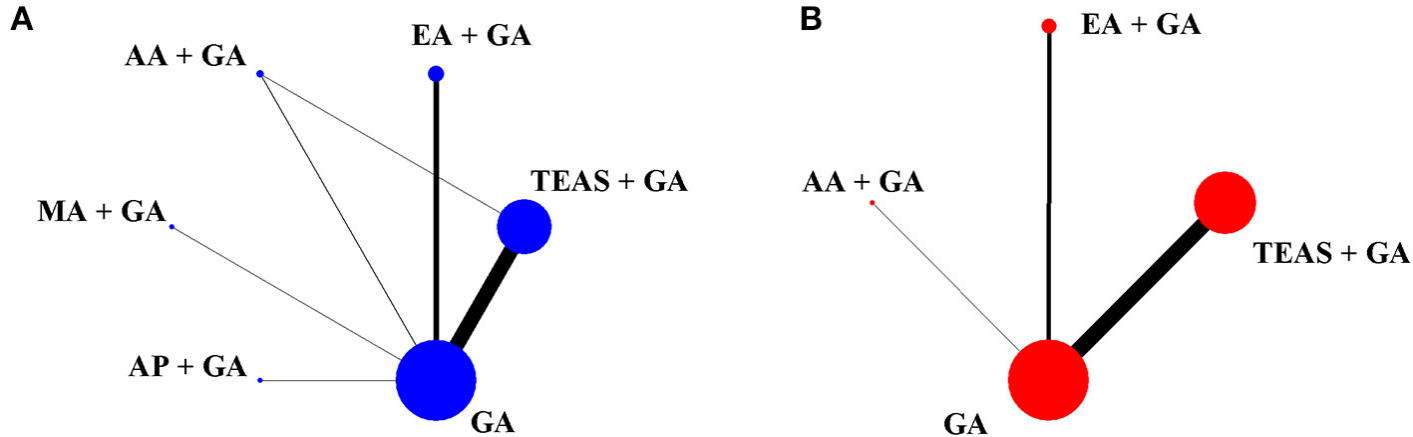
Network evidence plots for propofol **(A)** and remifentanil **(B)**. Straight lines connect interventions that were directly compared in RCTS that met the inclusion criteria. The size of each circular node is proportional to the sample size and the width of the lines represents the cumulative number of RCTs per pair of direct comparisons. TEAS, transcutaneous electrical acupoint stimulation; MA, manual acupuncture; EA, electroacupuncture; AA, auricular acupuncture; AP, acupressure; GA, general anesthesia.

As shown in Figure 1, 76 studies fulfilled the inclusion criteria (see Appendix 2 for all the references for included RCTs). A total of 6 treatments were included: TEAS-assisted GA (52 studies), EA-assisted GA (18 studies), AA-assisted GA (3 studies), MA-assisted GA (2 studies), AP-assisted GA (2 studies) and GA (76 studies). Definitions of each acupuncture technique are shown in Appendix 3. Of the studies, 98.7% (75/76) were two-arm and one three-arm. The propofol network evidence graph had 5 intervention nodes, forming a closed triangular loop: AA-assisted GA, TEAS-assisted and GA-GA. The remifentanil network evidence plot had 3 nodes with no triangular closed loops (Figure 2). Overall, 5,877 patients were included in the analyses, of whom 4,752 and 3,359 patients contributed to the outcomes of propofol and remifentanil, respectively. The characteristics of the included 76 RCTs are shown in Appendix 4. The years of publication ranged from 1993 to 2021. The mean age, weight and operation time of the patients was 51.28 years (SD = 8.74), 63.29 kg (SD = 9.85), and 119.38 min (SD = 30.22). Of the 76 included RCTs, 62 reported total doses of propofol (mg), 47 total doses of remifentanil (μg), and 33 both.

### 3.2. Risk of bias assessment

Regarding the quality of the included research articles, a randomization process was appropriately described in 11.8% of the trials (allocation concealment was not described in most of them). Of the trials, 53.9% had a low risk of deviations from the intended interventions. As the two outcomes were objective indicators, knowing the interventions that the subjects received did not affect outcome measures. Therefore, measurement of the outcome was at low risk of bias in all studies. The bias for missing outcome data was also low due to the fact that the included studies had no lost follow-ups or a low rate of lost follow-ups (< 5%). Of the trials, 13.2% were at low risk of selection of the reported result (for the remaining 86.8% there were some concerns since no experimental protocols were available) (see Appendix 5 for the risk of bias assessment). Overall, there was a moderate risk of bias across the entire network of evidence.

### 3.3. Pairwise meta-analysis results

**Figure 3:**
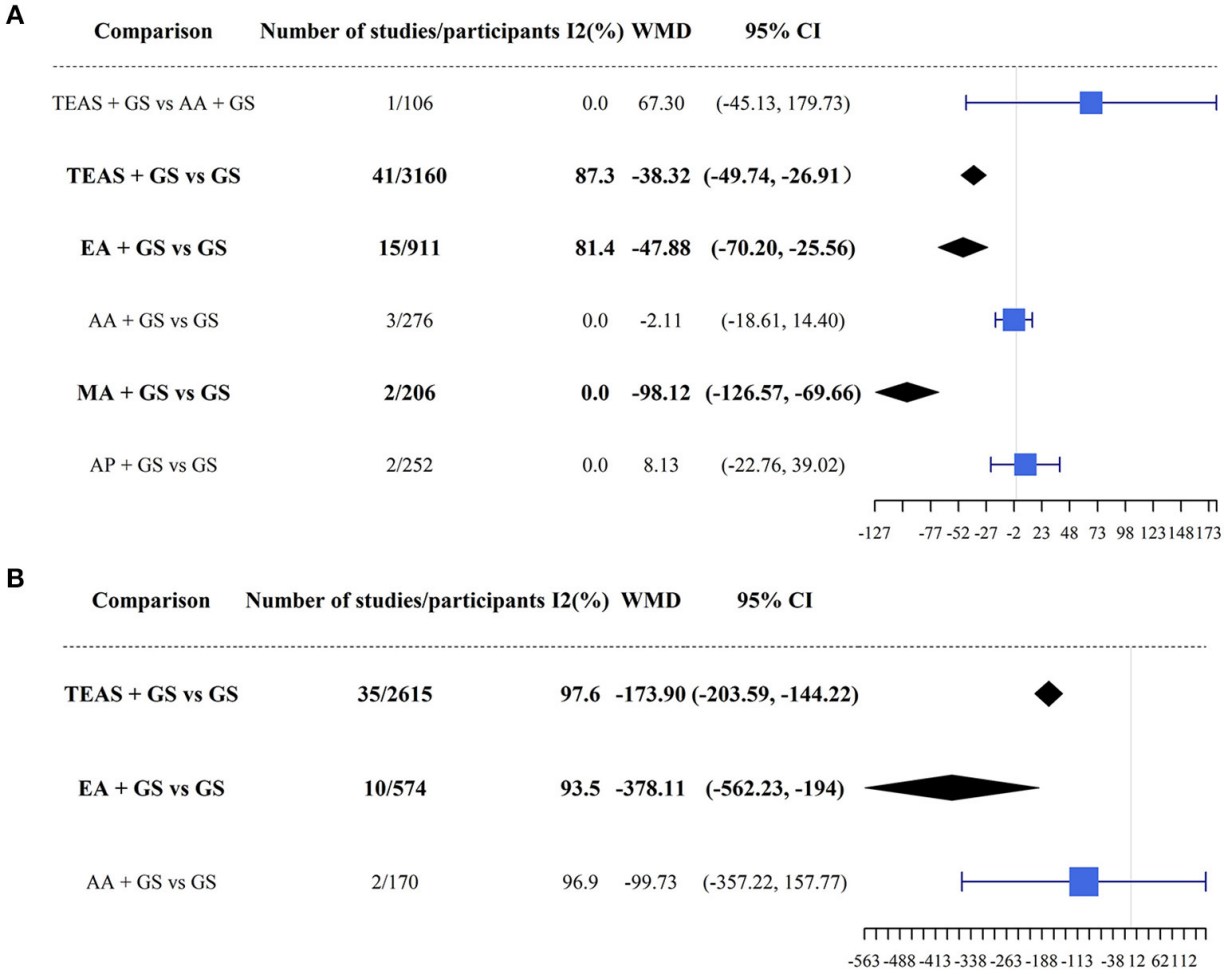
Results of direct pairwise meta-analysis for propofol **(A)** and remifentanil **(B)**. TEAS, transcutaneous electrical acupoint stimulation; MA, manual acupuncture; EA, electroacupuncture; AA, auricular acupuncture; AP, acupressure; GA, general anesthesia.

TEAS + GA (41 studies and 3,160 patients, WMD = −38.32, 95% CI [−49.74, −26.91], *I*^2^ = 87.3), EA + GA (15 studies and 911 patients, WMD = −47.88, 95% CI [−70.20, −25.56], *I*^2^ = 81.4), and MA + GA (2 studies and 206 patients, WMD = −98.12, 95% CI [−126.57, −69.66], *I*^2^ = 0) all achieved a lower total dosage of propofol than GA (*P* < 0.05) (Figure 3A).

TEAS + GA (35 studies and 2,615 patients, WMD = −173.90, 95% CI [−203.59, −144.22], *I*^2^ = 97.6) and EA + GA (10 studies and 574 patients, WMD = −378.11, 95% CI [−562.23, −194.00], *I*^2^ = 93.5) required less remifentanil total dosage during surgery than GA (*P* < 0.05) (Figure 3B).

### 3.4. Network meta-analysis results

The DICs of the two outcomes were 252.50 (propofol) and 187.76 (remifentanil) under the random effects model and 459.99 (propofol) and 1680.81 (remifentanil) under the fixed effect model. The random effect model was considered to have a better fit, so it was selected as the model for subsequent data analysis.

**Figure 4:**
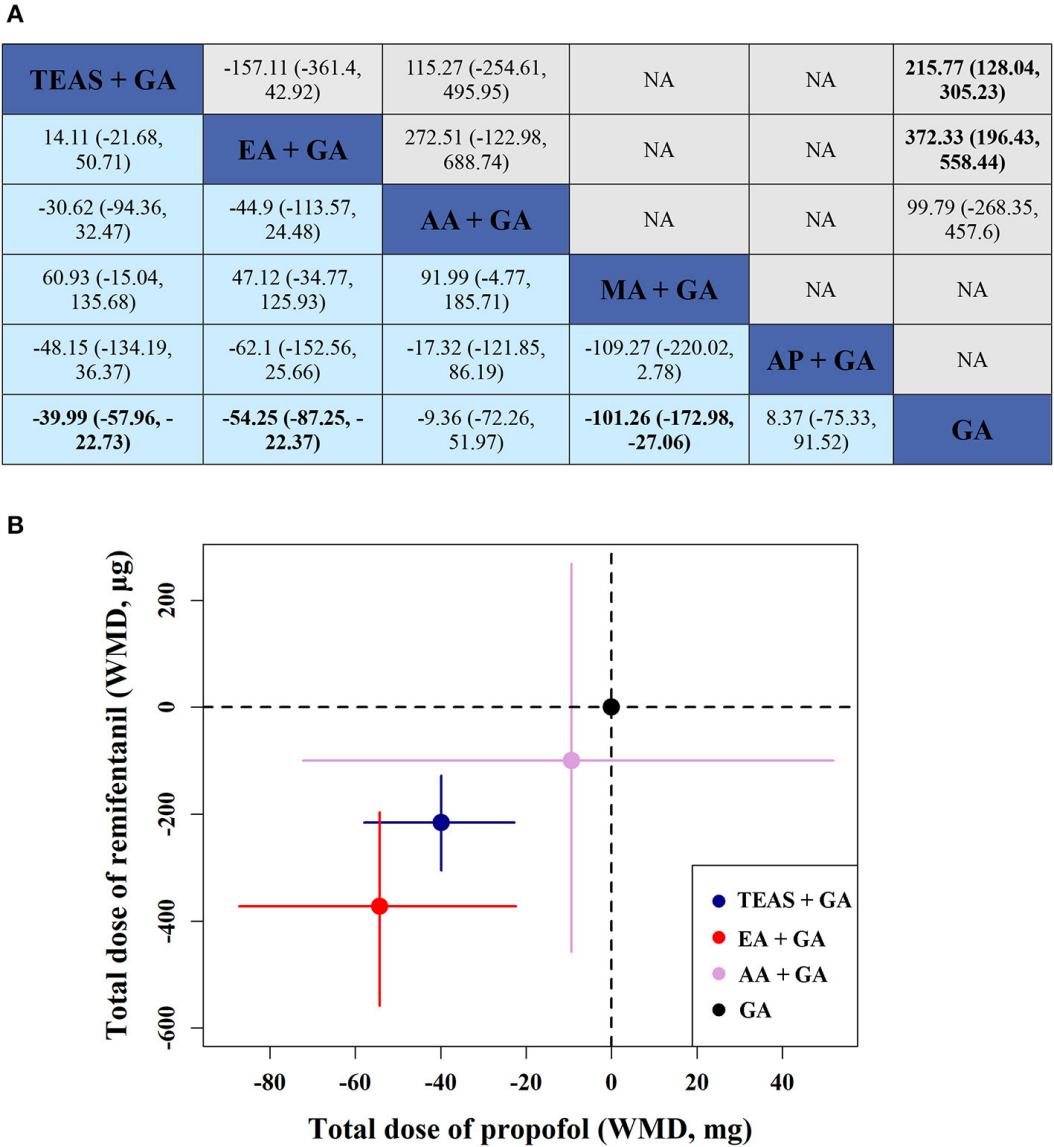
**(A)** WMD (weighted mean difference) with 95% CI of network meta-analysis for propofol and remifentanil. Results of network meta-analysis for propofol and remifentanil are shown in the lower and upper triangle, and the estimation was calculated as the column-defining treatment compared with the row-defining treatment. NA: not available. **(B)** Two-dimensional graphs about the total doses of propofol and remifentanil in network analysis. Data are reported as WMD in comparison with GA, which is the reference. Error bars are 95% CI. Individual acupunctures are represented by different colored nodes. TEAS, transcutaneous electrical acupoint stimulation; MA, manual acupuncture; EA, electroacupuncture; AA, auricular acupuncture; AP, acupressure; GA, general anesthesia.

The total intraoperative propofol volume was described in 62 studies that involved 4,752 patients. The results of the network meta-analysis revealed that the total propofol dosage of TEAS + GA (WMD = −39.99, 95% CI [−57.96, −22.73]), EA + GA (WMD = −54.25, 95% CI [−87.25, −22.37]) and MA + GA (WMD = −101.26, 95% CI [−172.98, −27.06]) were significantly lower than that of GA. Pairwise comparisons between other groups revealed no significant differences (*P* > 0.05) (Figure 4A, lower triangle).

Forty-seven studies that involved 3,359 patients reported the total intraoperative remifentanil dosage. The results of the network meta-analysis showed that: TEAS + GA (WMD = −215.77, 95% CI [−305.23, −128.04]) and EA + GA (WMD = −372.33, 95% CI [−558.44, −196.43]) required a significantly lower remifentanil dosage than GA. There was no difference in pairwise comparisons between the other groups (*P* > 0.05) (Figure 4A, upper triangle).

Based on network contribution plots (Appendix 6), a comparison of TEAS + GA vs. GA, EA + GA vs. GA, AA + GA vs. GA, MA+ GA vs. GA and AP + GA vs. GA appeared to contribute similarly to the entire network of propofol (roughly 20%). In the remifentanil network, the comparison of TEAS + GA vs. GA provided the largest contribution (57.0%).

Furthermore, it can be seen more intuitively in Figure 4B that EA + GA reduced the use of propofol by 14.26 mg and remifentanil by 156.56 μg on average, compared to TEAS + GA.

### 3.5. Evaluation of the results of transitivity, consistency and heterogeneity

Judging the transitivity by the box plots, it can be seen that the average age, weight and surgery time of patients in each comparison group of the propofol network were similar. The mean age and weight of each comparison group in the remifentanil network were also similar. However, the mean surgery time of EA + GA vs. GA was between 150 and 225 min, while that for TEAS + GA vs. GA and AA + GA vs. GA was between 60 and 150 min (Appendix 7).

The test for global inconsistency did not reveal any difference between the consistency and inconsistency models for propofol (*P* = 0.296). The test for local inconsistency showed that all loops were entirely consistent for propofol (*P* = 0.240). The inconsistency test evaluated by the node-splitting model revealed no significant differences between direct and indirect comparisons for propofol (Appendix 8).

The global *I*^2^ was 89.43% and 99.78% for propofol and remifentanil. Predictive interval plots indicated that 26.67% (4/15) and 33.33% (2/6) of the comparisons for propofol and remifentanil were affected to a large degree by the estimated heterogeneity in the network (see Appendix 9 for assessment of heterogeneity). The between-study heterogeneity τ derived from Bayesian meta-analysis was 47.02 for propofol and 254.02 for remifentanil.

From a visual point of view, the two funnel plots are roughly symmetrical, but there are individual studies that deviate from the central axis in the two figures. According to the color, they are direct comparison studies of TEAS + GA vs. GA and EA + GA vs. GA. The above findings did not indicate any significant risk of small sample effects or publication bias in the network (Appendix 10 for funnel plots).

### 3.6. SUCRA and the ranking of treatments

**Figure 5:**
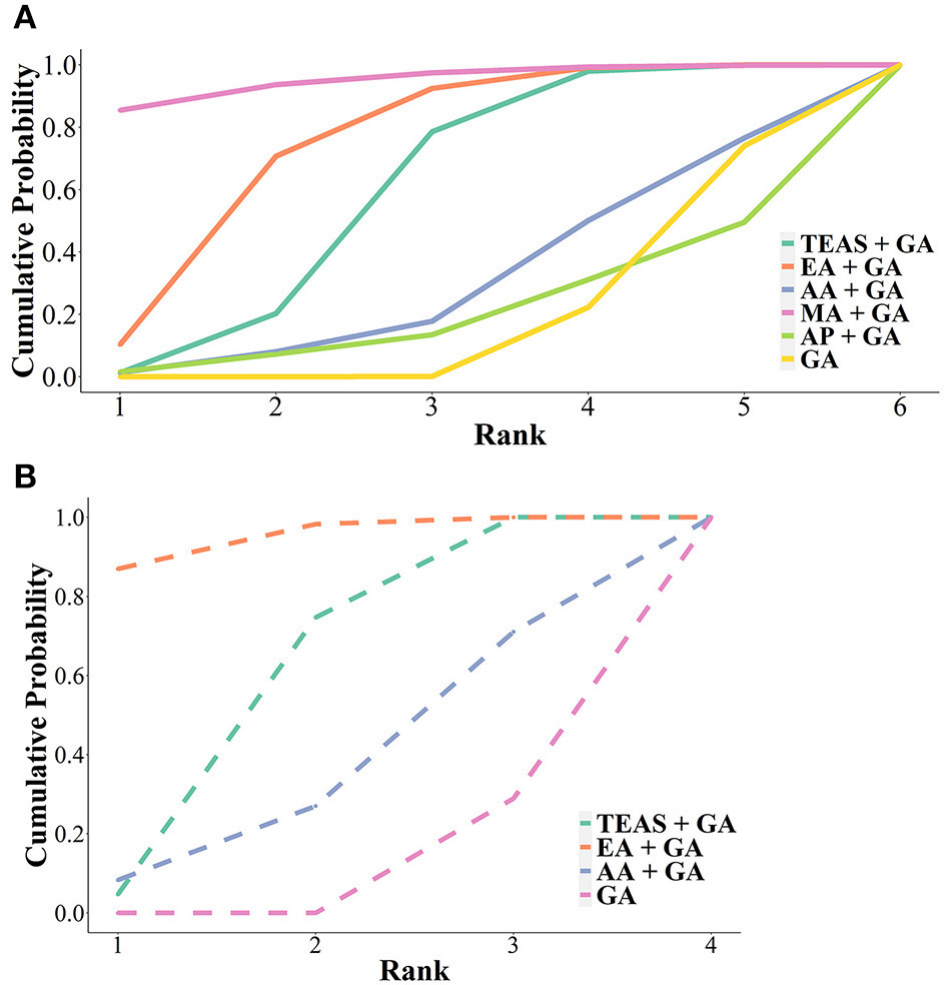
Cumulative ranking probability diagram of total amount of **(A)** propofol and **(B)** remifentanil under general anesthesia assisted by different acupuncture techniques. TEAS, transcutaneous electrical acupoint stimulation; MA, manual acupuncture; EA, electroacupuncture; AA, auricular acupuncture; AP, acupressure; GA, general anesthesia.

In terms of the efficacy of reducing the total dosage of propofol required during surgery, according to SUCRA the ranking probability of the 6 interventions from the first to the sixth was MA + GA (0.85), EA + GA (0.60), TEAS + GA (0.58), AA + GA (0.32), GA (0.52) and AP + GA (0.50) (Figure 5A).

In terms of the efficacy in reducing the total dosage of intraoperative remifentanil, according to SUCRA the ranking probabilities of the 4 interventions from the first to the fourth were EA + GA (0.87), TEAS + GA (0.70), AA + GA (0.44) and GA (0.71) (Figure 5B).

### 3.7. GRADE evaluation of evidence quality

The quality of evidence was rated to be low or moderate for all comparisons. In terms of TEAS + GA vs. GA and EA + GA vs. GA, the quality was moderate for propofol but low for remifentanil. For MA + GA vs. GA, the quality was moderate for propofol. Thus the evidence quality was moderate for the overall ranking of treatment with both propofol and remifentanil (see Appendix 11 for a contribution summary of the risk of bias assessment and Appendix 12 for the quality of evidence according to the GRADE framework).

### 3.8. Sensitivity analyses and subgroup analyses

Sensitivity evaluation of the network meta-analysis was carried out by using trials specifically designed to assess the effect of acupuncture-assisted GA on anesthesia. It confirmed the lower total propofol dosage of TEAS + GA, EA + GA and MA + GA vs. GA, as well as the lower total remifentanil dosage of TEAS + GA and EA + GA vs. GA, which were in good agreement with those previously found (Appendix 13).

Subgroup network meta-analyses for the total dosage of propofol compared with GA revealed the lowering of total dose of propofol during TEAS + GA was more evident in patients < 60 years old, adopting the method of TIVA, receiving abdominal or orthopedic surgery, and with a surgery duration < 120 min. The lowered propofol dosage combined with EA + GA was more evident in patients < 60 years old, adopting the method of TIVA, receiving abdominal surgery, with a surgery duration < 120 min, when administered before the induction of anesthesia and with ≤ 3 acupoints.

Subgroup network meta-analyses for the total dose of remifentanil compared to GA showed that a reduction in the total dosage of remifentanil combined with TEAS + GA was more evident in patients < 60 years old, when administered before the induction of GA until the completion of surgery, who received abdominal, mastectomy or neck surgery and a surgery duration < 120 min. The lowered total dosage of remifentanil when combined with EA + GA was more evident in patients < 60 years old, adopting the method of TIVA and those who experienced a surgery duration ≥120 min (Appendix 14).

## 4. Discussion

Traditional anesthesia usually uses sufficient sedative and analgesic drugs to ensure a satisfactory anesthetic effect, but accompanying adverse reactions such as injection pain, myoclonus, nausea and vomiting inevitably occur (13). There have been recent reports that general anesthetics, including inhalation anesthetics and opioids, may adversely affect cancer prognosis, such as increased postoperative recurrence or enhanced metastasis (14, 15). Therefore, a safe and tolerable assisted method is desperately needed to achieve adequate anesthesia at the lowest possible doses to minimize side effects in clinical practice. A 2015 meta-analysis involving three acupuncture techniques (EA, TEAS, AA) assisted GA demonstrated that AAA could reduce the total intraoperative propofol dosage required (MD = −59.29, 95% CI [−91.92, −26.67]). On this basis, we incorporated more RCT studies and acupuncture techniques, observed two indicators of common anesthetics propofol and remifentanil, conducted a network meta-analysis of 76 trials involving 5,877 patients and found that MA-assisted GA ranked first in reducing the total amount of propofol needed. Both EA- and TEAS-assisted GA significantly reduced the required propofol and remifentanil dosage compared to conventional GA, and EA-assisted GA was superior to TEAS-assisted GA in ranking the efficacy of reducing the total amount of propofol and remifentanil administered.

Acupuncture has a history of nearly 2,000 years in China. Traditional acupuncture, namely MA can be used to treat diseases by inserting needles into acupoints and lifting, inserting and twisting them. In order to perform quantitative and reproducible manipulation of acupuncture, EA and TEAS came into being. EA and TEAS are currently widely used to quantify the amount of stimulation produced by acupuncture. Using currents at a certain frequency and intensity, different depths of acupoints can be stimulated, and the amount of stimulation can be controlled objectively, including current intensity, stimulation frequency, stimulation time, etc. Both EA and TEAS save much manpower compared to MA. Moreover, TEAS is non-invasive and easily accepted by patients. To the best of our knowledge, the present study is the first network meta-analysis that has focused on the effects of MA-, EA-, and TEAS-assisted GA on the total intraoperative propofol dosage administered. All three methods significantly reduced the total dosage of intraoperative propofol required, and the efficacy of MA was the best technique. A recent review (6) summarized three potential mechanisms of perioperative acupuncture, namely effects on the autonomic nervous system, the inflammatory response and the endocannabinoid system. Multiple experimental studies on animals have demonstrated that EA can increase vagal nerve activity and inhibit sympathetic nerve activity (16, 17). EA has been shown to regulate the levels of TNF-A, IL-1B, IL-6, and myeloperoxidase, increase the levels of superoxide dismutase and reduce the levels of inflammatory proteins (18, 19). EA has been demonstrated to enhance the tolerance to acute cerebral ischemia-reperfusion injury and to attenuate cerebral injury by regulating the endocannabinoid system (20).

Opioids can cause respiratory depression, prolong hospital stays, impair cognitive functions, drug abuse and addiction in patients while providing precise analgesic actions (21). Therefore, non-opioid drugs or other technical means can be used in combination with opioids to achieve the target analgesic effect and reduce the dose of opioids required, thus reducing the incidence of dose-related side effects. This study found that both EA- and TEAS-assisted GA can significantly reduce the amount of intraoperative remifentanil administered. The scientific effectiveness of acupuncture for analgesia have been widely recognized at home and now abroad (22–24). The afferent fibers involved in acupuncture signaling and their interaction with pain signal afferent fibers have been the research hotspots of acupuncture analgesia (25). The acupoints affecting local pain have the same sensory afferent pathway as the pain site, and elicit a superior analgesic effect (26). Acupuncture can directly affect analgesia by stimulating different peripheral sensory afferent fibers (27). The class C nerve fiber reflex is one of the direct and objective indicators for evaluating pain and acupuncture analgesia (28, 29). Experiments on animals (30) have shown that the inhibitory effect of EA on the C-type nerve fiber reflex was significantly better than that of TEAS. In the present study, we found that the efficacy ranking of EA in reducing the total amount of remifentanil administered was superior to TEAS, which may be related to the fact that EA not only activated the surface, but also the deeper sensory afferents.

This is the first network meta-analysis that has comprehensively searched and analyzed the intraoperative anesthesia dosage required for all acupuncture techniques-assisted GA vs. conventional GA across an entire network of moderate quality. Furthermore, we carried out sensitivity analyses by including trials specifically designed to assess the effect of acupuncture-assisted GA on anesthesia dosage. The results were consistently significant, which indicated that our findings were robust. This study mainly focuses on intervention measures. However, there are many specific parameters of acupuncture techniques, so we conducted a detailed subgroup network meta-analysis according to the characteristics of each study (age, anesthesia method, comparison with sham acupuncture + GA or GA, the number of acupoints selected, the timing of administration, the type of surgery and surgery duration) to address study heterogeneity. In addition, the evaluation of evidence quality was incorporated into the GRADE framework for results interpretation.

However, a number of limitations of the present study should be pointed out. First, considering that RCTs with MA-assisted GA as interventions accounted for a low proportion (2/62) of all the included studies in the propofol network, and the subgroup network meta-analysis found that the efficacy of MA-assisted GA was more pronounced in elderly patients undergoing abdominal surgery, the interpretation of the results needs to be treated with caution. Second, the study only focused on the intraoperative anesthetic drug dosage required, without estimating patient prognosis-related outcomes such as postoperative drug doses, length of hospital stays, postoperative nausea and vomiting or pain scores, which will be investigated in future research to explore which kind of AAA technique is the best. Third, since acupuncture-assisted GA has not been standardized, the research baseline of various studies is inevitably inconsistent. The results therefore should be interpreted with caution despite our detailed subgroup analysis. However, we believe our study provides useful evidence for the standardization of acupuncture-assisted GA. Finally, some comparison groups in the GRADE framework were considered to be low-quality due to heterogeneity and indirectness, which may limit the general applicability of the results.

## 5. Conclusions

The most effective reduction of the total amount of propofol required for GA has been achieved by MA-assistance. Compared with conventional GA, both EA- and TEAS-assisted GA significantly reduced the total amount of propofol and remifentanil required during surgery, with EA producing greater reductions in these two outcomes than TEAS. EA appears to be an ideal acupuncture technique for surgical patients under GA in terms of reducing the dosages of the intraoperative anesthetic agents required, findings which should be incorporated in future clinical guidelines.

## Data availability statement

The original contributions presented in the study are included in the article/Supplementary material, further inquiries can be directed to the corresponding author.

## Author contributions

QR and CL were responsible for the conception and design of the study. MQ and TS were responsible for analysis and interpretation. QR, CL, MQ, and TS were responsible for acquisition and analysis of data, drafted the manuscript, read, and approved the final version of the manuscript. All authors contributed to the article and approved the submitted version.
