# Supplementary material for: Effect of perioperative acupuncture-assisted general anesthesia on the anesthetic dosage required in adult surgical patients: a network meta-analysis of randomized controlled trials
Source: Front Med (Lausanne). 2023 May 10;10:1133585. doi: 10.3389/fmed.2023.1133585 (PMC10206013; doi:10.3389/fmed.2023.1133585)
Supplement: Supplementary file 1 [file Data_Sheet_1.docx]

**Supplementary appendix to the manuscript**

## Appendix 1

## Search strategy and results

**Table S1. Number of citations from each database and the trial registers searched**

| **Databases and trial registers** | **Citations** |
| --- | --- |
| **English databases** |  |
| PubMed | 353 |
| Embase | 628 |
| Cochrane Library | 458 |
| Web of Science | 205 |
| **Chinese databases** |  |
| CBM | 572 |
| CNKI | 526 |
| Wanfang | 1,201 |
| VIP | 484 |
| **Total databases** | **4,427** |

**Search strategy for PubMed**

1. (acupuncture) OR (acupoint)) OR (acupoint stimulation)) OR (acupressure)) OR (acupoint injection)) OR (electroacupuncture)) OR (scalp acupuncture)) OR (auricular acupuncture)) OR (transcutaneous electrical stimulation)) OR (transcutaneous electrical acupoint stimulation) OR (transcutaneous electrical nerve stimulation) OR (ear acupuncture)

2. (general anesthesia) OR (intravenous anesthesia) OR (inhalational anesthesia)

3. (randomized controlled trial) OR (controlled clinical trial) OR (randomized) OR (placebo) OR (drug therapy) OR (randomly) OR (trial) OR (groups)

4. (animal [Title/Abstract]) OR (mice [Title/Abstract]) OR (rat [Title/Abstract]) OR (rats [Title/Abstract]) OR (animals [Title/Abstract]) OR (rabbit* [Title/Abstract])

5. (acupuncture) OR (acupoint) OR (acupoint stimulation)) OR (acupressure) OR (acupoint injection) OR (electroacupuncture) OR (scalp acupuncture)) OR (auricular acupuncture)) OR (transcutaneous electrical stimulation)) OR (transcutaneous electrical acupoint stimulation)) OR (transcutaneous electrical nerve stimulation) OR (ear acupuncture) AND (general anesthesia) OR (i.v. anesthesia) OR (inhalational anesthesia) AND (randomized controlled trial) OR (controlled clinical trial) OR (randomized) OR (placebo) OR (drug therapy) OR (randomly) OR (trial) OR (groups) NOT (animal [Title/Abstract]) OR (mice [Title/Abstract]) OR (rat [Title/Abstract]) OR (rats [Title/Abstract]) OR (animals [Title/Abstract]) OR (rabbit * [Title/Abstract]).

**Search strategy for Cochrane Library**

#1 (acupuncture) OR (acupoint) OR (acupoint stimulation) OR (acupressure) OR (acupoint injection) (Word variations have been searched)

#2 (electroacupuncture) OR (scalp acupuncture) OR (auricular acupuncture) OR (transcutaneous electrical stimulation) OR (transcutaneous electrical acupoint stimulation) (Word variations have been searched)

#3 (transcutaneous electrical nerve stimulation) OR (ear acupuncture) (Word variations have been searched)

#4 (general anesthesia) OR (intravenous anesthesia) OR (inhalational anesthesia) (Word variations have been searched)

#5 #1 OR #2 OR #3

#6 (randomized controlled trial) OR (controlled clinical trial) OR (randomized) OR (placebo) OR (drug therapy) (Word variations have been searched)

#7 (randomly) OR (trial) OR (groups) (Word variations have been searched)

#8 #6 OR #7

#9 (animal): ti, ab, kw OR (rat): ti, ab, kw OR (mice): ti, ab, kw OR (rabbit): ti, ab, kw (Word variations have been searched)

#10 #4 AND #5 AND #8 NOT #9

#11 #4 AND #5 AND #8

**Search strategy for Embase**

#1 acupuncture

#2 acupoints

#3 acupoints AND stimulation

#4 acupressure

#5 acupoint AND injection

#6 electroacupuncture

#7 scalp AND acupuncture

#8 auricular AND acupuncture

#9 transcutaneous AND electrical AND stimulation

#10 transcutaneous AND electrical AND acupoint AND stimulation

#11 transcutaneous AND electrical AND nerve AND stimulation

#12 ear AND acupuncture

#13 #1 OR #2 OR #3 OR #4 OR #5 OR #6 OR #7 OR #8 OR #9 OR #10 OR #11 OR #12

#14 general AND anesthesia

#15 intravenous AND anesthesia

#16 inhalational AND anesthesia

#17 #14 OR #15 OR #16

#18 randomized AND controlled AND trial

#19 controlled AND clinical AND trial

#20 randomized

#21 placebo

#22 drug AND therapy

#23 randomly

#24 trial

#25 groups

#26 #18 OR #19 OR #20 OR #21 OR #22 OR #23 OR #24 OR #25

#27 #13 AND #17 AND #26

#28 animal: ti, ab, kw OR rat: ti, ab, kw OR mice: ti, ab, kw OR rabbit: ti, ab, kw OR rats :ti, ab, kw OR rabbits: ti, ab, kw OR animals: ti, ab, kw

#29 #27 NOT #28

**Search strategy for Web of Science**

1 (ALL=(acupuncture) OR ALL=(acupoint) OR ALL=(acupoint stimulation) OR ALL=(acupressure)) OR ALL=(acupoint injection)) OR ALL=(electroacupuncture) OR ALL=(scalp acupuncture)) OR ALL=(auricular acupuncture) OR ALL=(transcutaneous electrical stimulation) OR ALL=(transcutaneous electrical acupoint stimulation) OR ALL=(transcutaneous electrical nerve stimulation) OR ALL=(ear acupuncture)

2 ((ALL=(general anesthesia) OR ALL=(intravenous anesthesia) OR ALL=(inhalational anesthesia)

3 (ALL=(randomized controlled trial )) OR ALL=(controlled clinical trial)) OR ALL=(randomized)) OR ALL=(placebo)) OR ALL=(drug therapy)) OR ALL=(randomly)) OR ALL=(trial)) OR ALL=(groups)

4 (AB=(animal)) OR AB=(mice) OR AB=(rat) OR AB=(rats) OR AB=(animals) OR AB=(rabbit) OR AB=(rabbits) OR TI=(animal) OR TI=(mice) OR TI=(rat) OR TI=(rats)) OR TI=(animals) OR TI=(rabbit) OR TI=(rabbits)

5 #1 AND #2 AND #3

6 #1 AND #2 AND #3 NOT #4

**Search strategy for CBM**

"穴位按压"[不加权:扩展]) OR "经皮神经电刺激"[不加权:扩展]) OR "灸法"[不加权:扩展]) OR "针刺疗法"[不加权:扩展]) OR "电针"[不加权:扩展]) OR "埋针"[不加权:扩展]) OR "针刺"[不加权:扩展]) OR "针灸疗法"[不加权:扩展]) OR "头针"[不加权:扩展]) OR "温针疗法"[不加权:扩展]) OR "耳针"[不加权:扩展]) OR "穴位贴敷法"[不加权:扩展]) OR "皮肤针疗法"[不加权:扩展]) OR ("穴位按压"[摘要:智能] OR "经皮神经电刺激"[摘要:智能] OR "灸法"[摘要:智能] OR "针刺疗法"[摘要:智能] OR "电针"[摘要:智能] OR "埋针"[摘要:智能] OR "针刺"[摘要:智能] OR "针灸疗法"[摘要:智能] OR "温针疗法"[摘要:智能] OR "头针"[摘要:智能] OR "耳针"[摘要:智能] OR "穴位贴敷"[摘要:智能] OR "皮肤针"[摘要:智能]))) AND ((("全身麻醉"[摘要:智能] OR "全麻"[摘要:智能]) )))) AND ((("随机对照"[摘要:智能] OR "随机"[摘要:智能] OR "RCT"[摘要:智能]) OR ("随机对照试验"[不加权:扩展]))))) NOT ((("动物"[摘要:智能] OR "兔"[摘要:智能] OR "鼠"[摘要:智能] OR "羊"[摘要:智能] OR "狗"[摘要:智能] OR "犬"[摘要:智能] OR "猴"[摘要:智能] OR "猪"[摘要:智能]) OR ("动物"[不加权:扩展]))))

**Search strategy for CNKI**

(TKA = '经皮穴位电刺激' OR TKA = '经皮神经电刺激" OR TKA = '穴位按压' OR TKA = '穴位按摩' OR TKA = '穴位埋线' OR TKA = '灸法' OR TKA = '穴位注射' OR TKA = '针灸疗法' OR TKA = '皮肤针' OR TKA = '平衡针' OR TKA = '穴位贴敷' OR TKA = '针灸' OR TKA = '电针' OR TKA = '针刺' OR TKA = '埋针' OR TKA = '头针' OR TKA = '耳针' OR TKA = '体针' OR TKA = '艾灸' OR TKA = '温针') AND (TKA = '全身麻醉' OR TKA = '全麻') AND ( TKA = '随机对照' OR TKA = '随机' OR TKA = 'RCT' ) NOT (TKA = '兔' OR TKA = '鼠' OR TKA = '羊' OR TKA = '狗' OR TKA = '犬' OR SU %= '动物' OR TKA = '动物') NOT (TKA = 'meta' OR TKA = 'Meta' OR TKA = '综述')

**Search strategy for Wanfang**

主题：（经皮穴位电刺激 or 经皮神经电刺激 or 穴位按压 or 穴位按摩 or 穴位埋线 or 灸法 or 穴位注射 or 针灸疗法 or 皮肤针 or 平衡针 or 穴位敷贴 or 针灸 or 电针 or 针刺 or 埋针 or 头针 or 耳针 or 体针 or 艾灸 or 温针） and 主题：（全身麻醉 or 全麻）and 主题：（随机对照 or 随机 or RCT）not 主题：（兔 or 鼠 or 狗 or 犬 or 羊 or 动物）not 主题：（meta or Meta or 综述）

**Search strategy for VIP**

(M = (经皮穴位电刺激 OR 经皮神经电刺激 OR 穴位按压 OR 穴位按摩 OR 穴位埋线 OR 灸法 OR 穴位注射 OR 针灸疗法 OR 皮肤针 OR 平衡针 OR 穴位敷贴 OR 针灸 OR 电针 OR 针刺 OR 埋针 OR 头针 OR 耳针 OR 体针 OR 艾灸 OR 温针) OR R = (经皮穴位电刺激 OR 经皮神经电刺激 OR 穴位按压 OR 穴位按摩 OR 穴位埋线 OR 灸法 OR 穴位注射 OR 针灸疗法 OR 皮肤针 OR 平衡针 OR 穴位敷贴 OR 针灸 OR 电针 OR 针刺 OR 埋针 OR 头针 OR 耳针 OR 体针 OR 艾灸 OR 温针)) AND (M = (全身麻醉 OR 全麻) OR R = (全身麻醉 OR 全麻)) AND (M = (随机对照 OR 随机 OR RCT) OR R = (随机对照 OR 随机 OR RCT)) NOT (M = (兔 OR 鼠 OR 狗 OR 犬 OR 羊 OR 动物) OR R = (兔 OR 鼠 OR 狗 OR 犬 OR 羊 OR 动物)) NOT (M = (meta OR Meta OR 综述) OR R = (meta OR Meta OR 综述))

## Appendix 2

**References for the included trials**

1. Bai Y. Transcutaneous Electrical Acupoint Stimulation combined with general anesthesia on the patient ' s thyroid surgery and acupoint specificity [Master's degree], 2015.

2. Cai Q, Wu C, Zhang Z, et al. Effect of percutaneous acupoint electrical stimulation assisted anesthesia on heart rate variability of patients after lumbar internal fixation．. *Guangdong Medical Journal* 2021;42(8):940-44. doi: 10.13820/j.cnki.gdyx.20210271

3. Chen Y, Yao Y, Wu Y, et al. Transcutaneous electric acupoint stimulation alleviates remifentanil-induced hyperalgesia in patients undergoing thyroidectomy: a randomized controlled trial. *International journal of clinical and experimental medicine* 2015;8(4):5781-7. [published Online First: 2015/07/02]

4. Christensen PA, Rotne M, Vedelsdal R, et al. Electroacupuncture in anaesthesia for hysterectomy. *British journal of anaesthesia* 1993;71(6):835-8. doi: 10.1093/bja/71.6.835 [published Online First: 1993/12/01]

5. Ding F, Xue J, Liu Y, et al. Effect of electroacupuncture combined with general anesthesia on stress response in patients with open posterior lumbar spine surgery. *Journal of Clinical Anesthesiology* 2021;37(2)

6. Ding Y, Gu C, Shen L, et al. Effects of acupuncture on neur omuscular block of vecur onium and analgesia under general an esthesia. *Chinese Journal of Anesthesiology* 2012(06):762-65.

7. Duan C, Cui Z, Zhang X, et al. Effect of transcutaneous electrical acupoint stimulation on postoperative cognitive dysfunction in elderly patients. *Hainan Medical Journal* 2019;30(16):2071-74. doi: 10.3969/j.issn.1003-6350.2019.16.010

8. Duan C, Lu X. Effect of transcutaneous electrical acuppoint stimulation on blood coagulation function durning cesarean delivery delivery perioperative in pregnancy associated thrombocytopenia *Shaanxi Medical Journal* 2017;46(6)

9. Fan X, Zhang F, Huang L, et al. Effects of TEAS combined with general anesthesia versus epidural block combined with general anesthesia on recovery of patients undergoing laparoscopic radical resection of colorectal cancer. *Chinese Journal of Anesthesiology* 2018;38(9)

10. Gao P, Shao B, Diao Y, et al. Effect of transcutaneous electrical acupoint stimulation on catheter related bladder discomfort after ureteroscopic lithotripsy. *Chinese Acupuncture & Moxibustion* 2020;40(8)

11. Gao X. Cardioprotective Effects of Electro-acupuncture Preconditioning in coronary heart disease patients Undergoing Non cardiac surgery. Fourth Military Medical University, 2013.

12. Gu C, Shen L, Ding Y, et al. Influence of acupuncture at acupoints and non-acupoints on the perioperative analgesic effect in patients with laparoscopic cholecystectomy. *Chinese Acupuncture & Moxibustion* 2010(8)

13. He B, Yang B. Analgesic effect of transcutaneous electrical acupoint stimulation combined with target-controlled infusion in general anesthesia and its influence on cardiovascular system. *Chinese Acupuncture & Moxibustion* 2008;28(3)

14. Hu X, Li J, Jin Y, et al. Clinical Study on Preemptive Analgesia Effect of Transcutaneous Electrical Acupoint Stimulation during First Total Knee Arthroplasty in Elderly Patients with Osteoarthritis. *Shandong Journal of Traditional Chinese Medicine* 2021;40(7)

15. Hu X, Xie Y, Lu Z, et al. Application of percutaneous electrical acupoint stimulation analgesia in radical mastectomy of breast cancer. *Modern Medicine Journal of China* 2014;16(8):13-16. doi: 10.3969/j.issn.1672-9463.2014.08.003

16. Huang L, Zhang F, Xu C, et al. Efficacy of TEAS for general anesthesia preserving spontaneous breathing in patients undergoing thoracoscopic surgery. *Chinese Journal of Anesthesiology* 2019;39(8)

17. Ji X, Sun X, Chen Y, et al. Effect of transcutaneous electrical acupoint stimulation assisted venous general anesthesia on postoperative rehabilitation and complications in patients undergoing gynecological hysteroscopic surgery. *Modern Journal of Integrated Traditional Chinese and Western Medicine* 2020;29(5):553-56. doi: 10.3969/j.issn.1008-8849.2020.05.024

18. Jia B. The Effects of Anesthetics and Transcutanclus Electrical Acupoint Stimulation on Skin Dynorphin and Kappa Receptor Expression [Master's degree]. Guangzhou University of Chinese Medicine, 2011.

19. Jiang H. Effect of transcutaneous electrical acupoint stimulation on perioperative cellular immunity and insulin resistance in diabetic patients during perioperative period [Master's degree]. Nanchang University, 2021.

20. Jin W, Mo Y, Dai Q, et al. Impacts of transcutanclus electrical acupoint stimulation assisted general anesthesia on analgesia and hemodynamics in radical mastectomy. *Modern Chinese Doctor* 2020;58(34)

21. Jin W, Mo Y, Jiang Q, et al. Effect of Transcutaneous Electrical Acupoint Stimulation on the Postoperative Ｒecovery Quality and Long － term Survival Quality in Breast Cancer Patients Undergoing Radical Mastectomy. *Chinese Journal of Integrated Traditional and Western Medicine* 2020;40(11):1315-21.

22. Klein AA, Djaiani G, Karski J, et al. Acupressure wristbands for the prevention of postoperative nausea and vomiting in adults undergoing cardiac surgery. *Journal of cardiothoracic and vascular anesthesia* 2004;18(1):68-71. doi: 10.1053/j.jvca.2003.10.014 [published Online First: 2004/02/20]

23. Kong X, Zhou J, Fu G, et al. Health and economic evaluation of laparoscopic cholecystectomy with combined acupuncture and drug anesthesia. *Zhejiang Journal of Traditional Chinese Medicine* 2013;48(12):867-69.

24. Li M, Deng Q, Zhu X, et al. Application and safety of electroacupuncture combined with TCI target-controlled infusion in thoracotomy with one-lung ventilation for esophageal cancer. *Modernization of Traditional Chinese Medicine and Materia Medica-World Science and Technology* 2021:1-7.

25. Li X, Zou R, Zheng M, et al. Effect of electroacupuncture pretreatment on propofol induction dose and hemodynamics during induction of anesthesia in elderly patients with general anesthesia: A clinical study of 30 cases. *Jiangsu Journal of Traditional Chinese Medicine* 2021;53(05):66-68. doi: 10.19844/j.cnki.1672-397X.2021.05.024

26. Li Y. Effect of transcutaneous electrical acupoint stimulation on perioperative stress response in patients undergoing endoscopic sinus surgery [Master's degree]. Lanzhou University, 2021.

27. Liang D, Jin S, Huang L, et al. The Effect of Transcutaneous Electrical Acupoint Stimulation on Postoperative Catheter-Related Bladder Discomfort in Patients Undergoing Transurethral Resection of the Prostate. *Evidence-based complementary and alternative medicine : eCAM* 2021;2021:6691459. doi: 10.1155/2021/6691459 [published Online First: 2021/02/26]

28. Liu X, Li S, Wang B, et al. Intraoperative and postoperative anaesthetic and analgesic effect of multipoint transcutaneous electrical acupuncture stimulation combined with sufentanil anaesthesia in patients undergoing supratentorial craniotomy. *Acupuncture in medicine : journal of the British Medical Acupuncture Society* 2015;33(4):270-6. doi: 10.1136/acupmed-2014-010749 [published Online First: 2015/05/01]

29. Liu Y. Remifentanil Sparing Effect of TEAS With Single or Dual Acupoints in Patients Undergoing Radical Mastectomy: A Single Center, Randomized, Double-blinded, Controlled Clinical Trial [A master's degree]. Fourth Military Medical University, 2016.

30. Lu L, Zhu H. Efect of eIectroacupuncture combined with dezocine on postoperative hyperalgesia induced by remifentanil *Shanghai Journal of Acupuncture and Moxibustion* 2005(04):6-7. doi: 10.13460/j.issn.1005-0957.2005.04.004

31. Lu Z, Wang Q, Sun X, et al. Transcutaneous electrical acupoint stimulation before surgery reduces chronic pain after mastectomy: A randomized clinical trial. *Journal of clinical anesthesia* 2021;74:110453. doi: 10.1016/j.jclinane.2021.110453 [published Online First: 2021/07/17]

32. Mai S, Meng J, Wang W, et al. Effect of electroacupuncture pretreatment on general anesthesia and recovery quality in patients undergoing colorectal cancer surgery. *Journal of Ningxia Medical College* 2017;39(01):42-45. doi: 10.16050/j.cnki.issn1674-6309.2017.01.011

33. Meng X, Li J, Zhang J, et al. Effects of transcutaneous electrical acupoint stimulation on emergence agitation in patients undergoing thoracoscopic surgery. *Chinese Journal of Anesthesiology* 2022;42(2):147-50. doi: 10.3760/cma.j.cn131073.20211203.00205

34. Mi E, Gao J, Chen X, et al. Effects of transcutaneous electrical acupoint stimulation on quality of recovery during early period after laparoscopic cholecystectomy. *Chinese Acupuncture & Moxibustion* 2018;38(3):256-60. doi: 10.13703/j.0255-2930.2018.03.007

35. Pan F, Gong H, He B, et al. Effects of different acupoints and stimulation methods on the prevention and treatment of nausea and vomiting after breast surgery. *Journal of New Chinese Medicine* 2014;46(3)

36. Pan L, Yang Y, Shao J, et al. Effects of perioperative transcutaneous electrical acupoint stimulation on postoperative analgesia in patients undergoing shoulder arthroscopic surgery. *Chinese Acupuncture & Moxibustion* 2019;39(1)

37. Pei X, Zhou Z, Xu G. Effect of electroacupuncture for immune function of patients treated with laparoscopic radical recectomy for rectal cancer. *Chinese Acupuncture & Moxibustion* 2016;36(6)

38. Qian Z. Effect of transcutanclus electrical acuppoint stimulation on postoperative delirium in elderly patients with spinal surgery [Master's degree]. Soochow University, 2018.

39. Qu N, Wang X, Su Y. Clinical Efficacy and Safety Analysis of Acupuncture Combined with General Anesthesia in Elderly Hip Ｒeplacement. *Liaoning Journal of Traditional Chinese Medicine* 2019;46(04):836-38. doi: 10.13192/j.issn.1000-1719.2019.04.049

40. Que B, Tu Q, Shi J, et al. Effects of Transcutaneous Electrical Acupoint Stimulation on Systemic Inflammatory Response Syndrome of Patients after Percutaneous Nephrolithotomy: A Randomized Controlled Trial. *Evidence-based complementary and alternative medicine : eCAM* 2021;2021:5909956. doi: 10.1155/2021/5909956 [published Online First: 2021/08/24]

41. Si J, Xu L, Li G, et al. Adjuvant effect of transcutaneous electrical acupoint stimulation in propofol—fentanyl anesthesia in partial mastectomy. *Journal of Southern Medical University* 2009;29(10)

42. Song K. Effects of transcutaneous electrical acupoint stimulation on gastrointestinal function in patients undergoing laparoscopic gastrectomy [Master's degree]. Wannan Medical College, 2020.

43. Tian Q, Mai S, Meng J. The auxiliary effect of electroacupuncture stimulation on intraoperative anesthetic dosage and postoperative nausea and vomiting in patients with general anesthesia. *Journal of Ningxia Medical College* 2018;40(11):1279-83. doi: 10.16050/j.cnki.issn1674-6309.2018.11.009

44. Wang D, Fang X, Zhang K, et al. Effect of them transcutaneous electrical acupoint stimulation at Hegu and Neiguan on prevention of postoperative sore throat after thyroid gland lobectomy.. *Chinese Journal of Information on Traditional Chinese Medicine* 2016;23(12)

45. Wang J. Application of acupuncture assisted general anesthesia in elderly patients undergoing laparoscopic cholecystectomy. *China Health Care Nutrition* 2019;29(32):87.

46. Wang L, Lin J, Ji G, et al. Influence of transcutaneous electrical acupoint stimulation on the using dosage of remifentanil in breast surgery. *Clinical Research and Practice* 2020;5(11):18-19. doi: 10.19347/j.cnki.2096-1413.202011008

47. Wang X. Effect of transcutaneous electrical stimulation with different acupoints compatibility in patients undergoing laparoscopic cholecystectomy [Master's degree]. Hebei North University, 2020.

48. Wang Y, Huang X, Yang C, et al. Effect of Transcutaneous Electrical Acupoint Stimulation on Perioperative Anxiety of Patients Undergoing Breast Cancer Surgery *Chinese Journal of Anesthesiology* 2020;40(12):1431-35. doi: 10.3760/cma.j.cn131073.20200626.01205

49. White PF, Hamza MA, Recart A, et al. Optimal timing of acustimulation for antiemetic prophylaxis as an adjunct to ondansetron in patients undergoing plastic surgery. *Anesthesia and analgesia* 2005;100(2):367-72. doi: 10.1213/01.ane.0000144425.16116.0a [published Online First: 2005/01/28]

50. White PF, Zhao M, Tang J, et al. Use of a disposable acupressure device as part of a multimodal antiemetic strategy for reducing postoperative nausea and vomiting. *Anesthesia and analgesia* 2012;115(1):31-7. doi: 10.1213/ANE.0b013e3182536f27 [published Online First: 2012/04/17]

51. Wu D, Zou X, Zhang F, et al. Effect of Electroacupuncture Stimulation on Dosage of Propofol and Midazolam in Cardiac Valve Replacement. *Journal of Guizhou Medical University* 2018;43(9)

52. Wu D, Zou X, Zhang F, et al. Effect of electroacupuncture stimulation on analgesic drug dosage during cardiac valve replacement. *Guizhou Medical Journal* 2019;43(01):54-56.

53. Wu Q, Zhang M, Wang L, et al. Effect of Transcutaneous Acupoint Electronic Stimulation Combined with Target Controlled Infusion of Propofol on Efficiency of General Anesthesia for Craniotomy. *Acupuncture Research* 2013;38(03):229-33. doi: 10.13702/j.1000-0607.2013.03.015

54. Xie S. Application of Transcutaneous Electrical Acupoint Stimulation to Anesthesia for Orthopedic Surgery in the Elderly. *Shanghai Journal of Acupuncture and Moxibustion* 2020;39(05):579-83. doi: 10.13460/j.issn.1005-0957.2020.05.0579

55. Xie YH, Chai XQ, Wang YL, et al. Effect of electro-acupuncture stimulation of Ximen (PC4) and Neiguan (PC6) on remifentanil-induced breakthrough pain following thoracal esophagectomy. *Journal of Huazhong University of Science and Technology Medical sciences = Hua zhong ke ji da xue xue bao Yi xue Ying De wen ban = Huazhong keji daxue xuebao Yixue Yingdewen ban* 2014;34(4):569-74. doi: 10.1007/s11596-014-1317-x [published Online First: 2014/08/20]

56. Xing Z, Ren Y, Tao M, et al. Study on the value of transcutaneous acupoint electrical stimulation with general intravenous anesthesia in subtotal thyroidectomy surgery. *Modern Journal of Integrated Traditional Chinese and Western Medicine* 2012;21(19)

57. Xu M, Zhang Y, Pi Y, et al. Application of total intravenous anesthesia combined with transcutaneous electrical acupoint stimulation to radical resection of rectal cancer. *Journal of Enhanced Recovery After Surgery* 2021;4(4):153-56.

58. Xu X, Wong S, Yu Y, et al. Effects of Percutaneous Acupoint Electrical Stimulation Assisted Anesthesia on Stress Response and Delirium after Lumbar Surgery in the Elderly. *Chinese Medical Innovations* 2022;19(16):53-57. doi: 10.3969/j.issn.1674-4985.2022.16.012

59. Yan X, Han X, Xing Q, et al. Comparative study between electroacupuncture at Neima point and Neiguan (PC 6) and epidural nerve block for preemptive analgesia in patients undergoing thoracic surgery. *Chinese Acupuncture & Moxibustion* 2021;41(01):59-64. doi: 10.13703/j.0255-2930.20200121-k0001

60. Yan Y, Li Y, Wu X, et al. The Anesthesiological Value of Transcutaneous Acupoint Electronic Stimulation Combined with General Intravenous Anesthesia in Endoscopic Thyroidectomy Patients: a Clinical Study　*Chinese Journal of Integrated Traditional and Western Medicine* 2014;34(05):545-48.

61. Yang B, Zhao W, Liao M, et al. The analgesic effect of transcutaneous electrical acupoint stimulation during intravenous general anesthesia. *Guangdong Medical Journal* 2008(08):1261-62. doi: 10.13820/j.cnki.gdyx.2008.08.039

62. Yang J, Ni C. Application of transcutaneous electrical acupoint stimulation combined with intravenous general anesthesia in elderly patients with gastric cancer surgery. *Guangdong Medical Journal* 2019;40(17):2536-38,42. doi: 10.13820/j.cnki.gdyx.20191185

63. Yang L, Xiong L, Lu Z, et al. Influence of electroacupuncture precondition ing on early cognitive disturbance in patients undergoing card iac surgery. *Chinese Heart Journal* 2009;21(05):712-15. doi: 10.13191/j.chj.2009.05.118.yanglf.003

64. Yang X, Zhou R, Zhu Z, et al. Application of acupuncture assisted general anesthesia in elderly patients undergoing laparoscopic cholecystectomy. *Henan Traditional Chinese Medicine* 2018;38(11):1751-54. doi: 10.16367/j.issn.1003-5028.2018.11.0467

65. Yang Z, Zhang Y, Guo Y, et al. Clinical study of transcutaneous electrical acupoint stimulation combined with general anesthesia in laparoscopic ovarian cyst resection. *Shaanxi Journal of Traditional Chinese Medicine* 2015;36(06):725-27.

66. Yang Z, Zhang Y, Tong B, et al. Effect of TEAS Combined with General Anesthesia on Cognitive Function of Patients Undergoing Gynaecological Laparoscopy. *Journal of Shaanxi College of Traditional Chinese Medicine* 2015;38(05):38-42. doi: 10.13424/j.cnki.jsctcm.2015.05.015

67. Yu J, Qu P, Fan H, et al. Observation on the Analgesic Effect of Transcutaneous Electrical Acupoint Stimulation for Breast Radical Carcinoma Operation. *Acupuncture Research* 2010;35(1):43-46.

68. Yuan H, Li X, Zhao X, et al. Effect of percutaneous electrical acupoint stimulation combined with general anesthesia in gynecological laparoscopic surgery. *China Health Care Nutrition* 2021;31(25):51.

69. Zhang L, Cao C, Li J, et al. Influence of auricular point sticking on incidence of nausea and vomiting and analgesia effect after gynecological laparoscopy. *Chinese Acupuncture & Moxibustion* 2013;33(4) [published Online First: 20130903]

70. Zhang Q, Zhao C, An L, et al. Effect of transcutaneous electrical acupoint stimulation combined with general anesthesia on intraoperative and postoperative recovery in patients undergoing carotid artery stenting. *International Journal of Anesthesiology and Resuscitation* 2019;40(6):525-30. doi: 10.3760/cma.j.issn.1673-4378.2019.06.004

71. Zhang R, Su J, Dou F. Application effect of transcutaneous electrical acupoint stimulation combined with target-controlled infusion in breast surgery anesthesia. *Henan Traditional Chinese Medicine* 2015;35(09):2259-61. doi: 10.16367/j.issn.1003-5028.2015.09.0957

72. Zhang Y, Zhu L, Lou J, et al. The Application of Acupuncture Anesthesia in Obese Patients Undergo Elective laparoscopic Cholecystectomy. *Journal of Emergency in Traditional Chinese Medicine* 2015;24(6):1079-81. doi: 10.3969/j.issn.1004-745X.2015.06.049

73. Zhao W, Zhao X, Li J, et al. Clinical observation on controlling antihypertension with the general anesthesia of TEAS and anesthetics in endoscopic endonasal surgery. *Chinese Acupuncture & Moxibustion* 2015;35(12)

74. Zhao Y, Ding Y. The effects of different frequency transcutanclus electrical acupoint stimulation on enhanced recovery after surgery of thyroid surgery under general anesthesia. *World Latest Medicine Information* 2021;21(15):43-44,48. doi: 10.3969/j.issn.1671-3141.2021.15.015

75. Zheng X, Wan L, Gao F, et al. Effect of ear point embedding on plasma and effect site concentrations of propofol-remifentanil in elderly patients after target-controlled induction. *Chinese Acupuncture & Moxibustion* 2017;37(08):869-73. doi: 10.13703/j.0255-2930.2017.08.017

76. Zhou M. Clinical Research on the Analgesic Effect of Electroacupuncture Preemptive Analgesia during the Perioperative Period in Thoracic Surgery [Master's degree]. Henan University Of Science And Technology, 2017.

## Appendix 3

**Definitions of the acupuncture techniques included in this study**

**Table S2. Definitions of the acupuncture techniques included in this study**

| **Acupuncture techniques included in this study** | **Definitions** |
| --- | --- |
| Manual acupuncture (MA) | MA refers to treating patients by inserting thin, solid needles into acupuncture points (acupoint) on the skin. The needles are often manipulated by the practitioner, with the intention of eliciting the Deqi sensation (i.e.; a pain, achiness, stinging or dullness at the needle insertion site, which is an indicator that the acupuncture needle has been correctly placed). |
| **Electro-acupuncture (EA)** | **EA** is an acupuncture technique that applies small electrical currents to needles that have been inserted at specific points on the body. |
| Transcutaneous Electrical Acupoint Stimulation (TEAS) | TEAS is a non-invasive alternative to needle-based electro-acupuncture (EA). It combines the acupuncture and transcutaneous electrical nerve stimulation (TENS) by pasting the electrode piece on the acupoint instead of sticking the needles on the skin. |
| **Auricular acupuncture (AA)** | AA is the stimulation of **acupuncture** points on the external ear surface for the diagnosis and treatment of health conditions another areas of the body. |
| Acupressure (AP) | AP is a**form of touch therapy** that utilizes the principles of acupuncture and Chinese medicine. In acupressure, the same points on the body are used as in acupuncture, but are stimulated with finger pressure instead of with the insertion of needles. |

## Appendix 4

**Characteristics of the 76 studies included in the network meta-analysis.**

**Table S3. Characteristics of the 76 studies included in the network meta-analysis.**

| **Study ID** | **Control** | **Size** | **Types of acupuncture** | **Types of surgery** | **A nesthesia** | **Acupoints** | **Frequency/**  **intensity** | **Administration and Duration** | **Outcome** | **Baseline information** | | |
| --- | --- | --- | --- | --- | --- | --- | --- | --- | --- | --- | --- | --- |
|  |  |  |  |  |  |  |  |  |  | **Age (yrs)** | **Weight (kg)** | **Operation length (min)** |
| Zhao 2021 | Sham | 60 | TEAS | Thyroidectomy or subtotal thyroidectomy | CIIA | Bilateral Hegu (LI4), Neiguan (PC6), Zusanli (ST36) | 2/15 Hz in the dense-disperse mode | 30 min before induction of anesthesia to the end of the surgery | Remifentanil | 42.7 | NA | 83.6 |
| Liu 2016 | Sham | 91 | TEAS | Radical mastectomy | TIVA | Neiguan (PC6) and Danzhong (RN17) | 2/10 Hz in the dense-disperse mode /according to the maximal tolerance of patients | 30 min before induction of anesthesia | Remifentanil | 47.0 | NA | 110.05 |
| Wu 2019 | Blank | 40 | Electroacupuncture | Cardiac valve replacement under cardiopulmonary bypass | TIVA | Bilateral Hegu (PC6), Quchi  (LI11), Zusanli (ST36) and Yinlingquan  (SP09) | 2/10 Hz in the dense-disperse mode/1-2 mA | 20 min before induction of anesthesia to the end of the surgery | Remifentanil | 47.5 | 52.8 | NA |
| Fei 2016 | sham | 50 | Electroacupuncture | Laparoscopic radical resection of rectal cancer | CIIA | Zusanli (ST36), Sanyinjiao (SP6) | 2 Hz/50 Hz in the dense-disperse mode/ < 2mA | 15 min before induction of anesthesia to the end of the surgery | Remifentanil | 56.0 | 59.0 | 234.5 |
| Wang 2016 | Sham | 110 | TEAS | Thyroid lobectomy | TIVA | Bilateral Hegu (LI4), Neiguan (PC6) | 2/100 Hz in the dense-disperse mode/8-12mA | 30 min before induction of anesthesia to the end of the surgery | Remifentanil | 49.4 | NA | 96.5 |
| Mi 2018 | Sham | 100 | TEAS | Laparoscopic cholecystectomy | CIIA | Bilateral Hegu (LI4), Neiguan (PC6) Zusanli (ST36) and its lateral 2-inch non-acupoints | 2 Hz/100 Hz in the dense-disperse mode/  8–12 mA | 30 min before induction of anesthesia to the end of the surgery | Remifentanil + Remifentanil | 44.5 | 62.9 | 44.2 |
| Wang 2020 | Sham | 200 | TEAS | Mastectomy | TIVA | Bilateral Hegu (LI4), Neiguan (PC6) | 2/100 Hz in the dense-disperse mode/according to the maximal tolerance of patients | 30 min before induction of anesthesia to the end of the surgery | Remifentanil | 46.2 | 59.4 | NA |
| Xin 2012 | Blank | 60 | TEAS | Bilateral subtotal thyroidectomy | TIVA | Bilateral Hegu (LI4), Neiguan (PC6) | 2/100 Hz in the dense-disperse mode/8–12 mA | 20 min before induction of anesthesia to the end of the surgery | Remifentanil | 40.8 | 63.5 | 77.2 |
| Yang 2019 | Sham | 87 | TEAS | Gastric cancer surgery | TIVA | Bilateral Hegu (LI4), Neiguan (PC6), Zusanli (ST36) | 2/100 Hz /according to the maximal tolerance of patients | 30 min before induction of anesthesia to the end of the surgery | Remifentanil | 72.6 | NA | 191.1 |
| Yu 2010 | Blank | 60 | TEAS | Radical mastectomy for breast cancer | TIVA | Affected side: Hegu (LI4), Laogong (PC8), Neiguan (PC6), Waiguan (TE5) | 2 Hz/100 Hz in the dense-disperse mode/5–10mA | 30 min before induction of anesthesia to the end of the surgery | Remifentanil | NA | NA | NA |
| Duan 2019 | Sham | 80 | TEAS | Hip replacement | CIIA | Bilateral Hegu (LI4), Neiguan (PC6) | 2/200 Hz in the dense-disperse mode/according to the maximal tolerance of patients | 30 min before induction of anesthesia to the end of the surgery | Remifentanil | 77.0 | 66.5 | 144.5 |
| Gao 2013 | Blank | 68 | TEAS | Abdominal surgery | CIIA | Neiguan (PC6), Lieque (LU7), Yunmen (LU2) | 5–6 Hz/25–30 Hz in the dense-disperse mode/  2.34–6.24 mA | 30 minutes/ day for three consecutive days before surgery | Remifentanil | 68.6 | 62.8 | 189.1 |
| Chen 2015 | Sham | 59 | TEAS | Thyroidectomy | TIVA | Bilateral Hegu (LI4) and Neiguan (PC6) | 2/10 Hz in the dense-disperse mode/6–9 mA | 30 min before the induction of anesthesia | Remifentanil | 41.7 | 57.3 | 94.7 |
| Lu 2018 | Blank | 60 | Electroacupuncture | Laparoscopic cholecystectomy | CIIA | Bilateral Hegu (LI4), Neiguan (PC6) Zusanli (ST36) and Yanglingquan (GB34) | Before surgery：continuous wave (4 Hz)/ according to the maximal tolerance of patients; during surgery:  4 Hz/20 Hz in the dense-disperse mode | 30 min before induction of anesthesia to the end of the surgery | Remifentanil | 54.5 | NA | 57.5 |
| Zheng 2017 | Sham | 50 | Auricular Acupuncture | Operation of external abdominal hernia | TIVA | Ear aupoints: Fugugou, Gan, Pizhixia and Shenmen on the operation side | NA | 30 min before induction of anesthesia to the end of the surgery | Propofol + Remifentanil | 67.5 | NA | 121.7 |
| Yang 2015 | Sham | 60 | TEAS | Laparoscopic ovarian cyst resection | TIVA | Bilateral Neiguan (PC6), Zusanli (ST36) | 2–100 Hz in the dense-disperse mode/30–80 mA | 30 min before induction of anesthesia to the end of the surgery | Propofol + Remifentanil | NA | NA | NA |
| Xie 2020 | Blank | 92 | TEAS | Orthopedic surgery | TIVA | Hegu (LI4), Neiguan (PC6) | 2–100 Hz /according to the maximal tolerance of patients | 5 min before induction of anesthesia to the end of the surgery | Propofol | 69.0 | NA | NA |
| Yang 2018 | Blank | 146 | Manual acupuncture | Laparoscopic cholecystectomy | TIVA | Bilateral Hegu (LI4) and Taichong (LR3) | NA | 20-30 min before induction of anesthesia | Propofol | 67.0 | 64.7 | 51.3 |
| Qu 2019 | Blank | 86 | Electroacupuncture | Hip replacement | TIVA | Bilateral Hegu, (LI4), Neiguan (PC6), and Sanyinjiao (SP6) | 2–10 Hz /according to the maximal tolerance of patients | 30 min before induction of anesthesia | Propofol + Remifentanil | 71.6 | NA | NA |
| Xu 2022 | Blank | 70 | TEAS | Single-level lumbar Vertebrae surgery | TIVA | Bilateral Hegu (LI4), Zusanli (ST36), Neiguan (PC6) | 2 Hz/30 Hz in the dense-disperse mode/6–10 mA | 30 min before induction of anesthesia to the end of the surgery | Propofol + Remifentanil | 69.8 | NA | NA |
| Wang 2019 | Blank | 60 | Manual acupuncture | Laparoscopic cholecystectomy | TIVA | Bilateral Hegu (LI4) and Taichong (LR3) | NA | 30 min before induction of anesthesia | Propofol | 74.1 | NA | NA |
| Hu 2021 | Blank | 100 | TEAS | Primary total knee arthroplasty for osteoarthritis | CIIA | The operation side: Chongmeng(SP12), Neimadian,Biguan (ST31), Zusanli (ST36), Sanyinjiao (SP6), Xuehai (SP10) | 2/100 Hz in the dense-disperse mode/15–20 mA | 1 day before surgery and 30 min before induction of anesthesia | Propofol + Remifentanil | NA | NA | NA |
| Qian 2018 | Sham | 90 | TEAS | Decompression and internal fixation for lumbar disc herniation | CIIA | Bilateral Zusanli(ST36), Taichong (LR3), Hegu (LI4), Neiguan (PC6) | 2/100 Hz/0.1–1 mA (During anesthesia:  30 mA) | Three days before surgery: twice a day for 30 min; On the day of surgery: stimulate once 30 min before surgery | Propofol | 69.3 | NA | 131.0 |
| Li 2021 | Blank | 60 | Electroacupuncture | Joint replacement | TIVA | Baihui (DU20), Yintang (DU29), Shenting (DU24) | 2/100 Hz in the dense-disperse mode/4–6 mA | 30 min before induction of anesthesia | Propofol | 71.4 | 63.7 | NA |
| Zhao 2015 | Sham | 60 | TEAS | Endoscopic surgery requiring controlled hypotension | CIIA | Bilateral Hegu (LI4), Zusanli (ST36), Sanyinjiao (SP6), Quchi (LI11) | 2 Hz /100 Hz /3–5 ｍA | 30 min before induction of anesthesia to the end of the surgery | Propofol | 41.0 | 62.1 | 85.5 |
| Pan 2014 | Blank | 159 | Auricular Acupuncture & TEAS | Mastectomy | TIVA | TEAS: Bilateral Neiguan (PC6) and Quchi (LI11); AA: Bilateral Shenmen、Jiaogan、Naogan、Neifenmi | TEAS: 2–50 Hz in the dense-disperse mode/3–15 mA | before induction of anesthesia to the end of the surgery | Propofol | 37.9 | 54.6 | 90.1 |
| Wang 2020 | Blank | 70 | TEAS | Laparoscopic cholecystectomy | CIIA | Hegu (LI4), Neiguan (PC6), Zusanli (ST36), Sanyinjiao (SP6) | 2/100 Hz in the dense-disperse mode/according to the maximal tolerance of patients | 30 min before induction of anesthesia to the end of the surgery | Propofol + Remifentanil | 47.5 | NA | 52.5 |
| Zhou 2017 | Blank | 55 | Electroacupuncture | Radical resection of esophageal cancer | TIVA | Bilateral Neimadian and Neiguan (PC6) | Continuous wave: (4–100 times/min)/output voltage positive pulse > 12.5V, negative pulse > 20V, 250 ω load | 30 min before induction of anesthesia | Propofol + Remifentanil | 54.5 | 59.5 | 220.0 |
| Tian 2018 | Blank | 50 | Electroacupuncture | Posterior lumbar inter body fusion | TIVA | Baihui (DU20), Sishencong (Ex-HN01) and bilateral Hegu (LI4), Neiguan (PC6), Zusanli (ST36), | 2/100 Hz in the dense-disperse mode/according to the maximal tolerance of patients | One day before surgery、30 min before induction of anesthesia to the end of the surgery | Propofol + Remifentanil | 47.6 | NA | 173.3 |
| Wu 2018 | Blank | 40 | Electroacupuncture | Cardiac valve replacement under cardiopulmonary bypass | TIVA | Bilateral Hegu(LI4), Quchi (LI11), Zusanli (ST36), Yinlingquan (SP09) | 2/100 Hz in the dense-disperse mode/1–2 mA | 20 min before induction of anesthesia to the end of the surgery | Propofol | 47.7 | 52.8 | NA |
| Li 2022 | Blank | 60 | Electroacupuncture | Thoracotomy of esophageal carcinoma with one lung ventilation | CIIA | Bilateral Houxi (SI3), Zhigou (SJ06), Hegu (LI4), Neiguan (PC6) | Continuous wave: 2 Hz/ according to the maximal tolerance of patients | 30 min before induction of anesthesia to the end of the surgery | Propofol | 55.6 | 62.8 | 164.1 |
| Ding 2021 | Blank | 113 | Electroacupuncture | Posterior lumbar open surgery | TIVA | Bilateral Hegu (LI4), Neiguan (PC6) | 2/10 Hz in the dense-disperse mode/according to the maximal tolerance of patients | 30 min before induction of anesthesia | Propofol + Remifentanil | 53.8 | 65.5 | 151.6 |
| Yan 2021 | Blank | 40 | Electroacupuncture | Radical resection of esophageal cancer | TIVA | Bilateral Neimadian and Neiguan (PC6) | Continuous wave /according to the maximal tolerance of patients | 30 min before induction of anesthesia | Propofol + Remifentanil | 54.5 | 58.0 | 220.5 |
| Mai 2017 | Blank | 40 | Electroacupuncture | Open radical resection for colorectal cancer | TIVA | Right side: Zusanli (ST36), Shangjuxu (ST37), Xiajuxu (ST39) | 2 Hz/2–3 mA | One day before surgery, 30 min before induction of anesthesia | Propofol + Remifentanil | 50.8 | NA | 194.4 |
| Li 2021 | Sham | 64 | TEAS | Nasal endoscopic surgery | TIVA | Hegu (LI4), Neiguan (PC6) | 2 Hz in the dense-disperse mode/6–9 mA | 30 min before induction of anesthesia to the end of the surgery | Propofol + Remifentanil | 41.8 | 68.6 | 96.5 |
| Song 2020 | Sham | 50 | TEAS | Laparoscopic gastrectomy | TIVA | Bilateral Hegu (LI4), Neiguan (PC6) | 5 Hz/100 Hz in the dense-disperse mode/15 mA | 30 min before induction of anesthesia to the end of the surgery | Propofol + Remifentanil | 59.6 | 61.5 | 224.7 |
| Wu 2013 | Sham | 40 | TEAS | Craniotomy | TIVA | Bilateral Yuyao (EX-HN4), Taiyang (EX-HN5), Hegu (LI4), Quanliao (SI18), Fengchi (GB20) | 2 Hz/100 Hz in the dense-disperse mode/8–12 mA | 20 min before induction of anesthesia to the end of the surgery | Propofol | 48.5 | 58.2 | 276.5 |
| Duan 2017 | Sham | 80 | TEAS | Cesarean section for pregnancy-associated thrombocytopenia | TIVA | Bilateral Hegu (LI4), Neiguan (PC6) | 2 Hz/100 Hz in the dense-disperse mode/ according to the maximal tolerance of patients | 30 min before induction of anesthesia to the end of the surgery | Propofol + Remifentanil | 28.1 | 70.2 | 46.5 |
| Jin 2020 | Sham | 61 | TEAS | Radical mastectomy for breast cancer | TIVA | Bilateral Hegu (LI4), Neiguan (PC6), Zusanli (ST36), Sanyinjiao (SP6) | 2 /100 Hz in the dense-disperse mode/ 6–12 mA | 30 min before induction of anesthesia to the end of the surgery | Propofol + Remifentanil | 49.6 | 58.6 | 127.5 |
| Gao 2020 | Sham | 57 | TEAS | Ureteroscopic lithotripsy | TIVA | Guanyuan (CV4), Zhongji (CV3), Zusanli (ST36), Sanyinjiao (SP6) | 2 Hz/15 Hz in the dense-disperse mode/6–10 mA | 30 min before induction of anesthesia | Propofol + Remifentanil | 50.5 | 73.8 | 50.5 |
| Jiang 2021 | Sham | 90 | TEAS | Gastrointestinal surgery | TIVA | Hegu (LI4) and Zusanli (ST36), | 2 Hz/100 Hz in the dense-disperse mode/5–15mA | 30 min before induction of anesthesia | Propofol + Remifentanil | 64.0 | NA | 212.9 |
| Ji 2020 | Blank | 100 | TEAS | Gynecological hysteroscopic surgery | TIVA | Bilateral Neiguan (PC6) | Continuous wave: 2 Hz/ according to the maximal tolerance of patients | 30 min before induction of anesthesia | Propofol | 40.8 | NA | 30.6 |
| Cai 2021 | Sham | 70 | TEAS | Posterior lumbar internal fixation | TIVA | Bilateral Neiguan (PC6) and Zusanli (ST36) | 2/15 Hz, in the dense-disperse mode/according to the maximal tolerance of patients | 30 min before induction of anesthesia to the end of the surgery | Propofol + Remifentanil | 56.9 | 63.6 | 129.5 |
| He 2008 | Blank | 60 | TEAS | Mastectomy | TIVA | Operation side: Hegu (LI4), Neiguan (PC6) | 2/100 Hz in the dense-disperse mode/according to the maximal tolerance of patients | 30 min before induction of anesthesia to the end of the surgery | Propofol | 37.4 | 53.8 | NA |
| Zhang 2015 | Blank | 64 | TEAS | Mastectomy | TIVA | Affected side: Hegu (LI4), Laogong (PC8), Neiguan (PC6), Waiguan (TE5), bilateral Jianjing (GB21) | 2–100 Hz in the dense-disperse mode/according to the maximal tolerance of patients | 30 min before induction of anesthesia to the end of the surgery | Propofol | 42.2 | 52.9 | NA |
| Yan 2014 | Blank | 60 | TEAS | Bilateral subtotal thyroidectomy | TIVA | Bilateral Hegu (LI4), Neiguan (PC6) | 2/100 Hz in the dense-disperse mode/8–12 mA | 30 min before induction of anesthesia | Propofol | 38.5 | 57.0 | 121.0 |
| Pan 2019 | Sham | 80 | TEAS | Unilateral shoulder arthroscopic surgery | CIIA | Affected side: Hegu (LI4), Neiguan (PC6) | 2 Hz/100 Hz in the dense-disperse mode/according to the maximal tolerance of patients | 30 min before surgery | Propofol + Remifentanil | 58.0 | 66.3 | 85.0 |
| Zhang 2015 | Blank | 105 | Electroacupuncture | Cholecystectomy | CIIA | Bilateral Hegu (LI4), Neiguan (PC6)and Zusanli (ST36) | 2/100 Hz in the dense-disperse mode/15 mA | 30 min before induction of anesthesia | Propofol | 55.1 | NA | 64.4 |
| Gu 2010 | Blank | 90 | Electroacupuncture | Laparoscopic cholecystectomy | TIVA | Hegu (LI4), Neiguan (PC6), Zusanli (ST36) Yanglingquan (GB34) | 4 Hz/20 Hz, in the dense-disperse mode/ 5 mA | 15-30 min before induction of anesthesia | Propofol | 55.1 | NA | 61.9 |
| Fan 2018 | Blank | 52 | TEAS | Laparoscopic radical resection of colorectal cancer | CIIA | Bilateral Hegu (LI4), Neiguan (PC6), Zusanli (ST36) Shangjuxu (ST37), Xiajuxu (ST39) | 2/100 Hz in the dense-disperse mode/  3–8 mA | 30 min before induction of anesthesia to the end of the surgery | Propofol + Remifentanil | 54.0 | 58.0 | 176.0 |
| Huang 2019 | Blank | 40 | TEAS | Thoracoscopic surgery | CIIA | The operation side: Xinshu (BL15) and Feishu (BL13); Bilateral Hegu (LI4), Neiguan (PC6) | 2/100 Hz in the dense-disperse mode/5–15 mA | 30 min before induction of anesthesia to the end of the surgery | Propofol + Remifentanil | 46.0 | NA | 78.5 |
| Jia 2011 | Blank | 40 | TEAS | Modified radical mastectomy for unilateral breast cancer | CIIA | Hegu (LI4), Neiguan (PC6) on the opposite side of the operation; Bilateral Taichong (LR3) and Sanyinjiao (SP6) | 2/100 Hz in the dense-disperse mode/according to the maximal tolerance of patients | 30 min before induction of anesthesia to the end of the surgery | Propofol | 48.2 | 56.4 | 86.9 |
| Meng 2022 | Sham | 80 | TEAS | Thoracoscopic surgery | TIVA | Hegu (LI4), Zusanli (ST36) and Sanyinjiao (SP6) | 2/100 Hz, in the dense-disperse mode/3–8 mA | 30 min before induction of anesthesia to the end of the surgery | Propofol + Remifentanil | 57.0 | 68.0 | 116.0 |
| Xu 2021 | Blank | 40 | TEAS | Open radical resection of rectal cancer | TIVA | Bilateral Hegu (LI4), Neiguan (PC6) and Zusanli (ST36) | 2/100 Hz in the dense-disperse mode/7–11 mA | 30 min before induction of anesthesia to the end of the surgery | Propofol | 52.3 | 71.5 | 187.3 |
| Zhang 2019 | Sham | 82 | TEAS | Carotid artery stenting | TIVA | The surgical side: Shuigou (DU26), Baihui (DU20), Hegu (LI4), Waiguan (TE5) | 2 Hz/100 Hz in the dense-disperse mode/according to the maximal tolerance of patients | 30 min before induction of anesthesia to the end of the surgery | Propofol + Remifentanil | 59.5 | 72.0 | 63.0 |
| Si 2010 | Blank | 60 | TEAS | Unilateral mastectomy | TIVA | Affected side Neiguan (PC6), Waiguan (TE5), Hegu (LI4), Laogong (PC8); Bilateral Jianjing (GB21) | 2/100 Hz in the dense-disperse mode/according to the maximal tolerance of patients | 30 min before induction of anesthesia to the end of the surgery | Propofol | 42.3 | 53.0 | 32.1 |
| Hu 2014 | Sham | 60 | TEAS | Radical mastectomy for breast cancer | TIVA | Bilateral Hegu (LI4), Neiguan (PC6) and Zusanli (ST36) | 2 Hz/30 Hz in the dense-disperse mode/3–6 mA | 30 min before induction of anesthesia | Propofol + Remifentanil | NA | NA | NA |
| Yang 2008 | Blank | 100 | TEAS | Unilateral segmentectomy of the breast | TIVA | The surgical side: Hegu (LI4), Neiguan (PC6) | 2/100 Hz in the dense-disperse mode/according to the maximal tolerance of patients | 30 min before induction of anesthesia to the end of the surgery | Propofol | 35.3 | 52.4 | 30.5 |
| Yuan 2021 | Blank | 60 | TEAS | Gynecological laparoscopic surgery | TIVA | Hegu (LI4), Neiguan (PC6), Zusanli(ST36), Sanyinjiao (SP6) | 2:100 Hz/NA | 30 min before induction of anesthesia | Propofol + Remifentanil | 28.8 | 64.1 | NA |
| Ding 2012 | Blank | 60 | Electroacupuncture | Laparoscopic cholecystectomy | TIVA | Bilateral Hegu (LI4), Neiguan (PC6), Zusanli (ST36) Yanglingquan (GB34), Quchi (LI11) | 4 Hz/20 Hz in the dense-disperse mode/5 mA | 15-30 min before induction of anesthesia to the end of the surgery | Propofol | 55.0 | 63.0 | 62.0 |
| Bai 2015 | Sham | 60 | TEAS | Thyroid surgery | TIVA | Bilateral Futu (LI18), Tianding (LI17) | 2/100 Hz/ reduced by  1 mA from the patient's maximum tolerated current. | 30 min before induction of anesthesia to the end of the surgery | Propofol + Remifentanil | 46.0 | NA | 44.0 |
| Kong 2013 | Blank | 60 | Electroacupuncture | Laparoscopic cholecystectomy | TIVA | Bilateral Riyue (GB24), Qichong (ST30) and Yanglingquan (GB34) | 30 Hz in the continuous wave mode. The frequency and intensity were adjusted according to the time of operation, hemodynamic parameters and BIS value | 20 min before induction of anesthesia | Propofol | 53.0 | 63.1 | 30.5 |
| Wang 2021 | Sham | 84 | TEAS | Modified radical mastectomy for breast cancer | CIIA | Yintang (DU29); Neiguan (PC6), Lao Gong (PC8) and Zusanli (ST36) on the operation side | 2/100 Hz in the dense-disperse mode/< 15mA | One day before surgery, 30 min before induction of anesthesia | Propofol + Remifentanil | 49.5 | NA | 132.0 |
| Yang 2009 | Blank | 75 | Electroacupuncture | Coronary artery bypass grafting and heart valve replacement under cardiopulmonary bypass。 | TIVA | Bilateral Neiguan (PC6), Lieque (LU7), Yunmen (LU2) | 5–6 Hz/25–30 Hz/2.34–6.24 mA | 30 minutes a day for 5 consecutive days before surgery | Propofol | 50.8 | NA | 192.0 |
| Que 2021 | Sham | 60 | TEAS | Percutaneous Nephrolithotomy | TIVA | bilateral Shenshu (BL23), Yinlingquan (SP9), Hegu (LI4), and Neiguan (PC6) | 2/100 Hz in the dense-disperse mode (in which the frequency was automatically alternated at every 3 s between 2 and 100 Hz). | 30 min before anesthesia induction till the end of the surgery (at the intervals of 30 min). | Propofol + Remifentanil | 46.3 | 58.0 | 112.3 |
| Liang 2021 | Blank | 70 | TEAS | Benign prostatic hyperplasia surgery | TIVA | RN7, RN6, RN5, RN4, and RN3 and bilateral BL32, BL33, and BL34. | 2/100 Hz/the highest tolerable level that caused no discomfort to the patient; | 30 minutes before general anesthesia | Propofol + Remifentanil | 70.0 | 63.5 | 53.9 |
| Liu 2015 | Sham | 88 | TEAS | Supratentorial craniotomy | TIVA | five pairs of acupuncture points: Hegu (LI4) and Waiguan (TE5), Jinmen (BL63) and Taichong (LR3), Zusanli (ST36) and Qiuxu (GB 40), and Fengchi (GB20) with Tianzhu (BL10) and Cuanzhu (BL2) with Yuyao (EX-HN4) on the craniotomy side. | with a dense-disperse frequency of 2/100 Hz (alternated once every 3 s; 0.6 ms at 2 Hz and 0.2 ms at 100 Hz). The intensity of stimulation was set at 4.89 ± 2.15, 6.79 ± 3.51, 7.04 ± 3.35 and 5.61 ± 2.13, respectively, according to the maximal tolerance of patients | 30 min before anesthesia induction,  maintained throughout the operation and terminated  at the end of surgery | Propofol | 43.0 | 65.3 | 245.9 |
| Christensen 1993 | Blank | 50 | Electroacupuncture | Hysterectomy. | TIVA | GV2 = Govenor vessel 2, Jao-Iu; GV4 = Govenor vessel 4, Ming-men; B 32 = bladder 32, Ciliao; SP 6 = spleen 6, Sanjiniao; ST 36 = stomach 36, Zusanli | using a constant current source with pulse width 320 ms, approximately 12 V, and chain frequencies  10 Hz and  100 Hz | *2*0 min before skin incision and continued to the  end of surgery. | Propofol | 44.0 | 66.0 | 106.5 |
| Klein 2004 | Sham | 152 | Acupressure | primary coronary artery bypass graft or valvular surgery | CIIA | the P6 point | NA | after establishment of intravenous access and invasive monitoring, but before induction of anesthesia, and were removed  24 h after extubation | Propofol | 62.5 | 79.0 | 204.0 |
| Lu 2021 | Sham | 378 | TEAS | Radical mastectomy | TIVA | bilateral PC6  (Neiguan) and  CV17 (Danzhong). | The device provided  “disperse-dense” waves with alternating frequencies of 2 Hz and 10 Hz for 2 cycles. The stimulation intensity was identified as the maximal tolerance | 30 min before anesthesia induction | Propofol | 48.2 | NA | 123.5 |
| White 2005 | Sham | 70 | TEAS | plastic surgery  (e.g., abdominoplasty, breast reductions, or facial  cosmetic procedures) | CIIA | the P6 acupoint of the nondominant arm | NA | 30 min before entering the operating  room | Propofol | 40.5 | 69.0 | 161.5 |
| White 2012 | Sham | 100 | Acupressure | major laparoscopic (e.g., cholecystectomy, bariatric gastric banding) surgery procedures | CIIA | bilateral P6 point | NA | 30 to 60 min before entering the operating room | Propofol | 44.5 | 93.5 | 113.5 |
| Xie 2014 | Sham | 40 | Electroacupuncture | Elective radical esophagectomy | TIVA | The ipsilateral acupoints of Ximen (PC4) and Neiguan (PC6) | a low frequency of  2 Hz and a high frequency of 20 Hz; The intensity of the stimulation was adjusted to the submaximal level that could be tolerated by the patient. | 30 min prior to induction of general anesthesia and continued through the surgery. | Propofol + Remifentanil | 54.5 | 59.5 | 219.0 |
| Zhang 2013 | Sham | 120 | Auricular Acupuncture | Gynecological laparoscopy | TIVA | Ear acupoints: Shenmen, Wei, Jiaogan | NA | Before surgery; 1, 5, 9 and  23 h after returning to the ward after surgery | Propofol + Remifentanil | 34.5 | 60.0 | 77.0 |
| Yang 2015 | Sham | 60 | TEAS | Laparoscopic oophorocystectomy | TIVA | Bilateral Neiguan (PC6), Zusanli (ST36) | 2–100 Hz/  30–80 mA | 30 min before induction of anesthesia to the end of the surgery | Propofol + Remifentanil | NA | NA | NA |
| Jin 2020 | Sham | 80 | TEAS | Radical mastectomy for breast cancer | TIVA | Bilateral Hegu (LI4), Neiguan (PC6), Zusanli (ST36), Sanyinjiao (SP6) | 2 Hz/100 Hz /6–12 mA | 30 min before induction of anesthesia to the end of the surgery | Propofol + Remifentanil | 49.8 | 58.3 | 127.5 |

Note: CIIA: Combined intravenous and inhalation anesthesia; TIVA: total intravenous anesthesia; NA: not available. TEAS: transcutaneous electrical acupoint stimulation

## Appendix 5

## Risk of bias assessment

1. **Risk of bias summary: it is a summary table of review authors’ judgments for each risk of bias entry for each study**


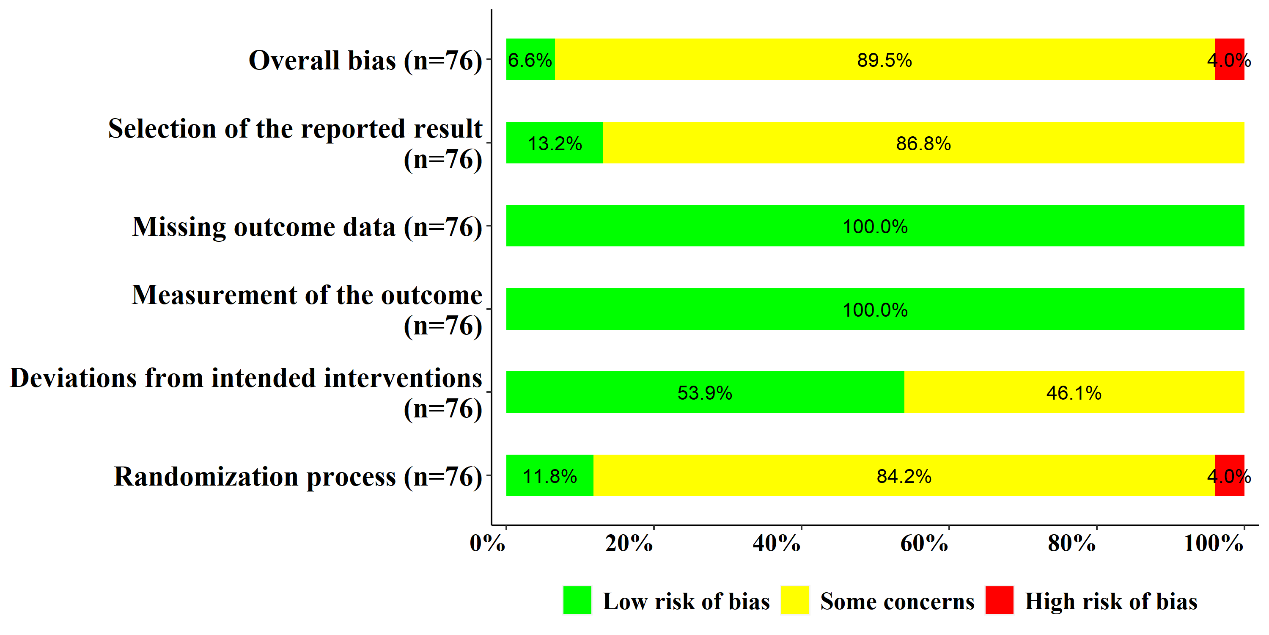


1. **Risk of bias table: it is the distribution of judgments (High; Some concerns; Low) across studies for each risk of bias entry**

| **Study ID** | **Randomization process** | **Deviations from intended interventions** | **Measurement of the outcome** | **Missing outcome data** | **Selection of the reported result** | **Overall bias** |
| --- | --- | --- | --- | --- | --- | --- |
| Zheng 2017 | Low | Low | Low | Low | Some concerns | Some concerns |
| Yang 2015 | Some concerns | Low | Low | Low | Some concerns | Some concerns |
| Xie 2020 | Some concerns | Low | Low | Low | Some concerns | Some concerns |
| Yang 2018 | Some concerns | Low | Low | Low | Some concerns | Some concerns |
| Qu 2019 | Some concerns | Low | Low | Low | Some concerns | Some concerns |
| Xu 2022 | Some concerns | Low | Low | Low | Some concerns | Some concerns |
| Wang 2019 | Some concerns | Low | Low | Low | Some concerns | Some concerns |
| Hu 2021 | Some concerns | Some concerns | Low | Low | Some concerns | Some concerns |
| Qian 2018 | Some concerns | Low | Low | Low | Some concerns | Some concerns |
| Li 2021 | Some concerns | Some concerns | Low | Low | Some concerns | Some concerns |
| Zhao 2015 | Some concerns | Low | Low | Low | Some concerns | Some concerns |
| Pan 2014 | Some concerns | Some concerns | Low | Low | Some concerns | Some concerns |
| Wang 2020 | Some concerns | Some concerns | Low | Low | Some concerns | Some concerns |
| Zhou 2017 | High | Some concerns | Low | Low | Some concerns | High |
| Tian 2018 | Some concerns | Some concerns | Low | Low | Some concerns | Some concerns |
| Wu 2018 | High | Some concerns | Low | Low | Some concerns | High |
| Li 2022 | Some concerns | Some concerns | Low | Low | Some concerns | Some concerns |
| Ding 2021 | Some concerns | Low | Low | Low | Low | Some concerns |
| Yan 2021 | High | Some concerns | Low | Low | Some concerns | High |
| Mai 2017 | Some concerns | Some concerns | Low | Low | Some concerns | Some concerns |
| Li 2021 | Low | Low | Low | Low | Some concerns | Some concerns |
| Song 2020 | Some concerns | Low | Low | Low | Some concerns | Some concerns |
| Wu 2013 | Some concerns | Low | Low | Low | Some concerns | Some concerns |
| Duan 2017 | Some concerns | Some concerns | Low | Low | Some concerns | Some concerns |
| Jin 2020 | Some concerns | Low | Low | Low | Some concerns | Some concerns |
| Gao 2020 | Some concerns | Low | Low | Low | Low | Some concerns |
| Jiang 2021 | Some concerns | Low | Low | Low | Low | Some concerns |
| Ji 2020 | Some concerns | Some concerns | Low | Low | Some concerns | Some concerns |
| Cai 2021 | Low | Low | Low | Low | Some concerns | Some concerns |
| He 2008 | Some concerns | Some concerns | Low | Low | Some concerns | Some concerns |
| Zhang 2015 | Some concerns | Some concerns | Low | Low | Some concerns | Some concerns |
| Yan 2014 | Some concerns | Some concerns | Low | Low | Some concerns | Some concerns |
| Pan 2019 | Some concerns | Low | Low | Low | Some concerns | Some concerns |
| Zhang 2015 | Some concerns | Some concerns | Low | Low | Some concerns | Some concerns |
| Gu 2010 | Some concerns | Some concerns | Low | Low | Some concerns | Some concerns |
| Fan 2018 | Some concerns | Some concerns | Low | Low | Some concerns | Some concerns |
| Huang 2019 | Some concerns | Some concerns | Low | Low | Some concerns | Some concerns |
| Jia 2011 | Some concerns | Some concerns | Low | Low | Some concerns | Some concerns |
| Meng 2022 | Some concerns | Some concerns | Low | Low | Some concerns | Some concerns |
| Xu 2021 | Some concerns | Some concerns | Low | Low | Some concerns | Some concerns |
| Zhang 2019 | Some concerns | Some concerns | Low | Low | Some concerns | Some concerns |
| Si 2010 | Some concerns | Some concerns | Low | Low | Some concerns | Some concerns |
| Hu 2014 | Some concerns | Low | Low | Low | Some concerns | Some concerns |
| Yang 2008 | Some concerns | Some concerns | Low | Low | Some concerns | Some concerns |
| Yuan 2021 | Some concerns | Some concerns | Low | Low | Some concerns | Some concerns |
| Ding 2012 | Some concerns | Some concerns | Low | Low | Some concerns | Some concerns |
| Bai 2015 | Some concerns | Low | Low | Low | Some concerns | Some concerns |
| Kong 2013 | Some concerns | Some concerns | Low | Low | Some concerns | Some concerns |
| Wang 2021 | Low | Low | Low | Low | Low | Low |
| Yang 2009 | Some concerns | Some concerns | Low | Low | Low | Some concerns |
| Que 2021 | Low | Low | Low | Low | Low | Low |
| Liang 2021 | Low | Low | Low | Low | Low | Low |
| Liu 2015 | Some concerns | Low | Low | Low | Low | Some concerns |
| Christensen 1993 | Some concerns | Some concerns | Low | Low | Some concerns | Some concerns |
| Klein 2004 | Some concerns | Low | Low | Low | Some concerns | Some concerns |
| Lu 2021 | Low | Low | Low | Low | Low | Low |
| White 2005 | Some concerns | Low | Low | Low | Some concerns | Some concerns |
| White 2012 | Some concerns | Low | Low | Low | Some concerns | Some concerns |
| Xie 2014 | Some concerns | Low | Low | Low | Some concerns | Some concerns |
| Zhang 2013 | Some concerns | Low | Low | Low | Some concerns | Some concerns |
| Yang 2015 | Some concerns | Low | Low | Low | Some concerns | Some concerns |
| Jin 2020 | Some concerns | Low | Low | Low | Some concerns | Some concerns |
| Zhao 2021 | Some concerns | Low | Low | Low | Some concerns | Some concerns |
| Liu 2016 | Low | Low | Low | Low | Low | Low |
| Wu 2019 | Some concerns | Some concerns | Low | Low | Some concerns | Some concerns |
| Fei 2016 | Some concerns | Low | Low | Low | Some concerns | Some concerns |
| Wang 2016 | Some concerns | Low | Low | Low | Some concerns | Some concerns |
| Mi 2018 | Some concerns | Low | Low | Low | Some concerns | Some concerns |
| Wang 2020 | Some concerns | Low | Low | Low | Some concerns | Some concerns |
| Xin 2012 | Some concerns | Some concerns | Low | Low | Some concerns | Some concerns |
| Yang 2019 | Some concerns | Low | Low | Low | Some concerns | Some concerns |
| Yu 2010 | Some concerns | Some concerns | Low | Low | Some concerns | Some concerns |
| Duan 2019 | Some concerns | Low | Low | Low | Some concerns | Some concerns |
| Gao 2013 | Some concerns | Some concerns | Low | Low | Some concerns | Some concerns |
| Chen 2015 | Low | Low | Low | Low | Some concerns | Some concerns |
| Lu 2018 | Some concerns | Some concerns | Low | Low | Some concerns | Some concerns |

## Appendix 6

## Contribution plots for the propofol and remifentanil network


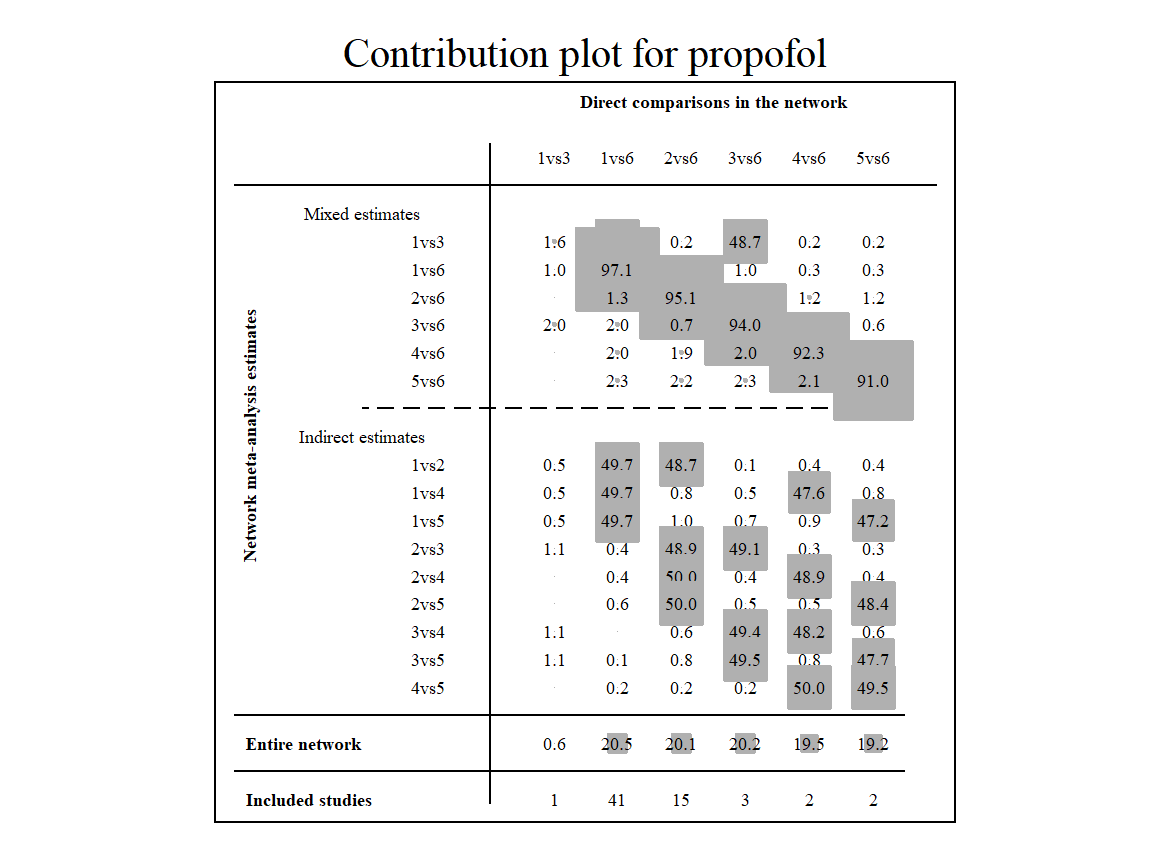


**Figure S1-1. Contribution plot of propofol. Note: The size of the squares is proportional to the percentage contribution of the column-defining direct comparison to the row-defining network estimate**

1:TEAS + GA; 2: EA + GA; 3: AA + GA; 4: MA + GA; 5: AP + GA; 6: GA. TEAS: transcutaneous electrical acupoint stimulation; MA: manual acupuncture; EA: electroacupuncture; AA: auricular acupuncture; AP: acupressure; GA: general anesthesia


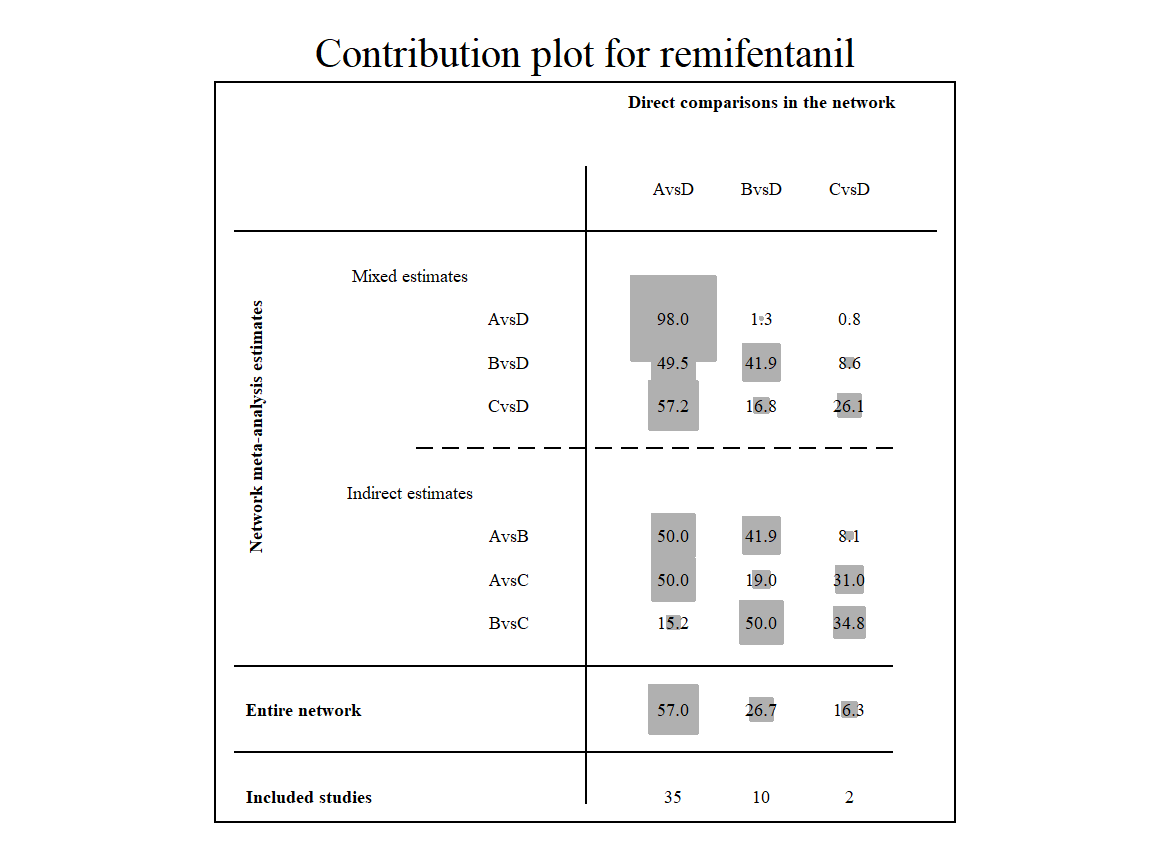


**Figure S1-2. Contribution plot of remifentanil. Note: The size of the squares is proportional to the percentage contribution of the column-defining direct comparison to the row-defining network estimate.**

A: TEAS + GA; B: EA + GA; C: AA + GA; D: GA. TEAS: transcutaneous electrical acupoint stimulation; EA: electroacupuncture; GA: general anesthesia.

## Appendix 7

## Assessment of transitivity results among 76 trials


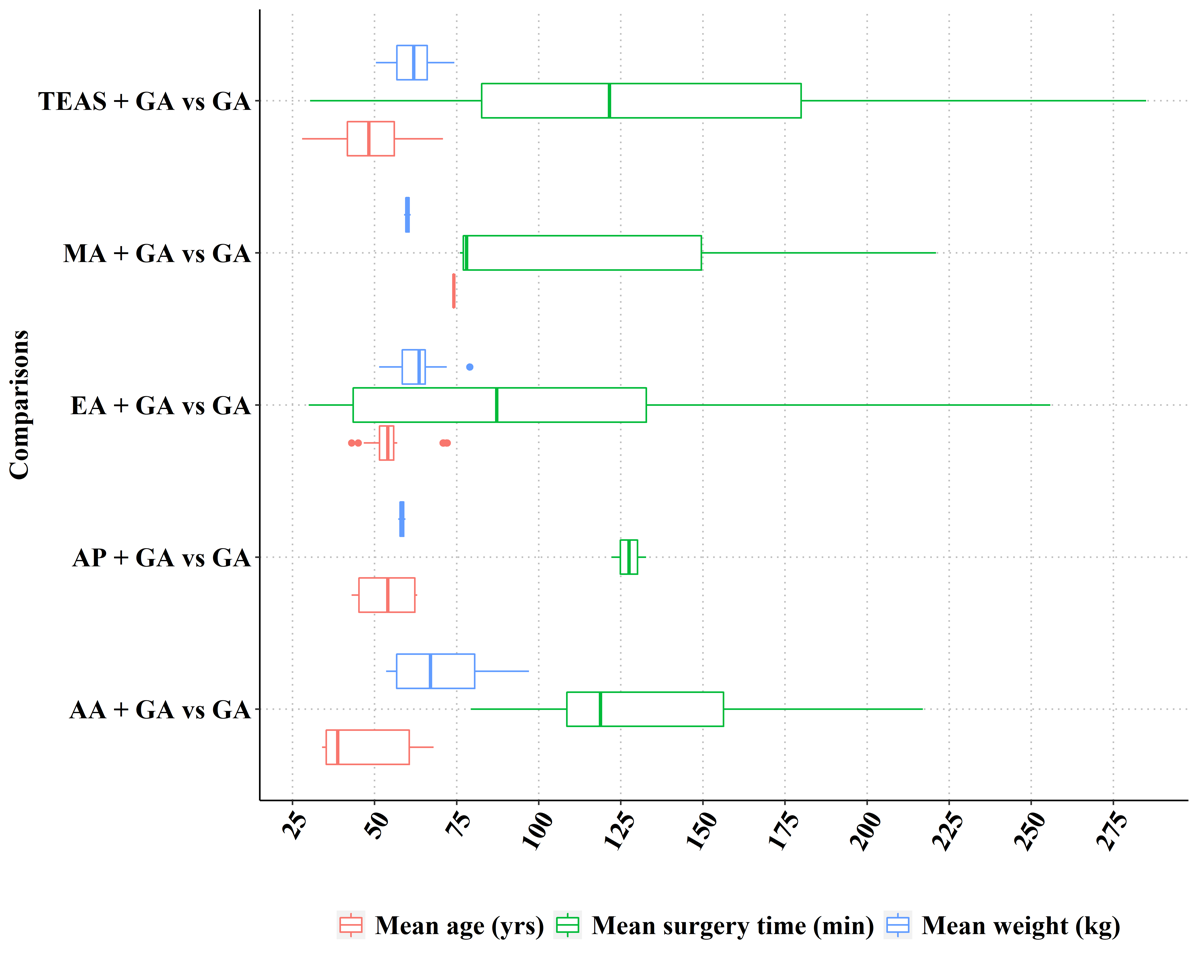


**Figure S2-1. Box plots among trials included for propofol**

TEAS: transcutaneous electrical acupoint stimulation; MA: manual acupuncture; EA: electroacupuncture; AA: auricular acupuncture; AP: acupressure; GA: general anesthesia


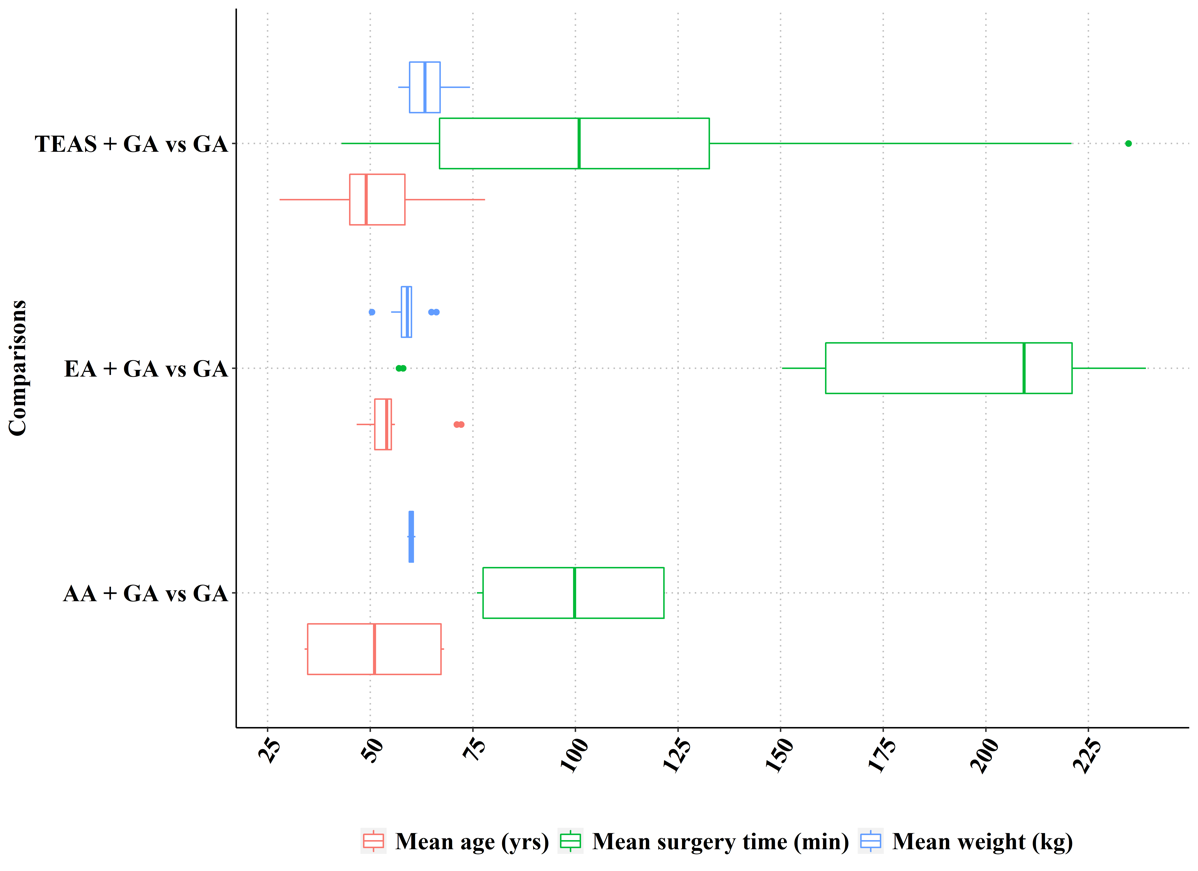


**Figure S2-2. Box plots among trials included for remifentanil**

TEAS: transcutaneous electrical acupoint stimulation; EA: electroacupuncture; GA: general anesthesia

## Appendix 8

**Assessment of inconsistency results: local and from the node-splitting model**

**a. Evaluation of the local inconsistency**


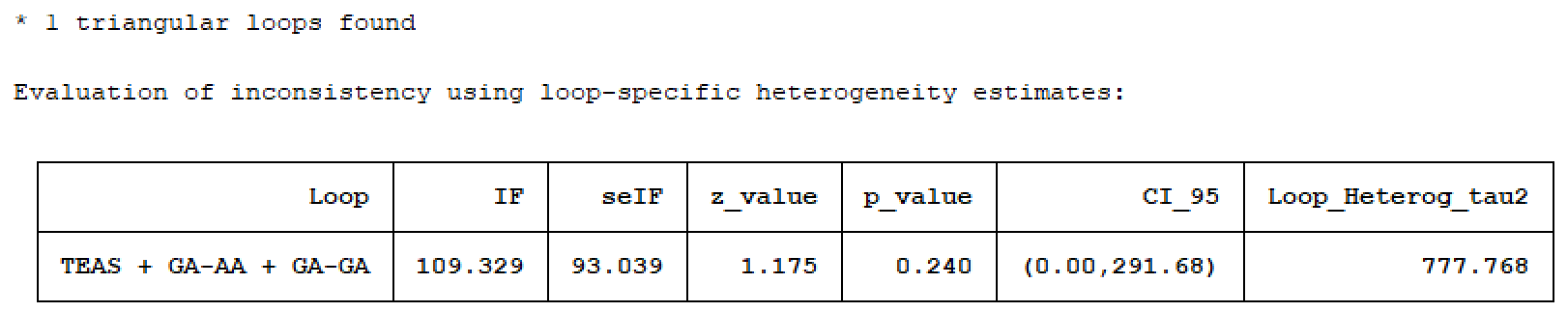


Note: TEAS: transcutaneous electrical acupoint stimulation; AA: auricular acupuncture; GA: general anesthesia

1. **Evaluation of the inconsistency by node-splitting model**

**Table S4. Propofol**

| **Comparisons** | **Direct** | | **Indirect** | | **Difference** | | |
| --- | --- | --- | --- | --- | --- | --- | --- |
|  | **MD** | **SE** | **MD** | **SE** | **MD** | **SE** | ***P*-value** |
| TEAS + GA VS GA | 40.54 | 8.53 | -88.51 | 140.82 | 129.05 | 141.06 | 0.360 |
| TEAS + GA VS AA + GA | -64.10 | 72.70 | 52.88 | 34.05 | -116.97 | 80.83 | 0.148 |
| AA + GA VS GA | -3.09 | 30.50 | 207.69 | 131.76 | -210.78 | 135.94 | 0.121 |

Note: TEAS: transcutaneous electrical acupoint stimulation; AA: auricular acupuncture; GA: general anesthesia

## Appendix 9

## Predictive intervals plot for the propofol and remifentanil network


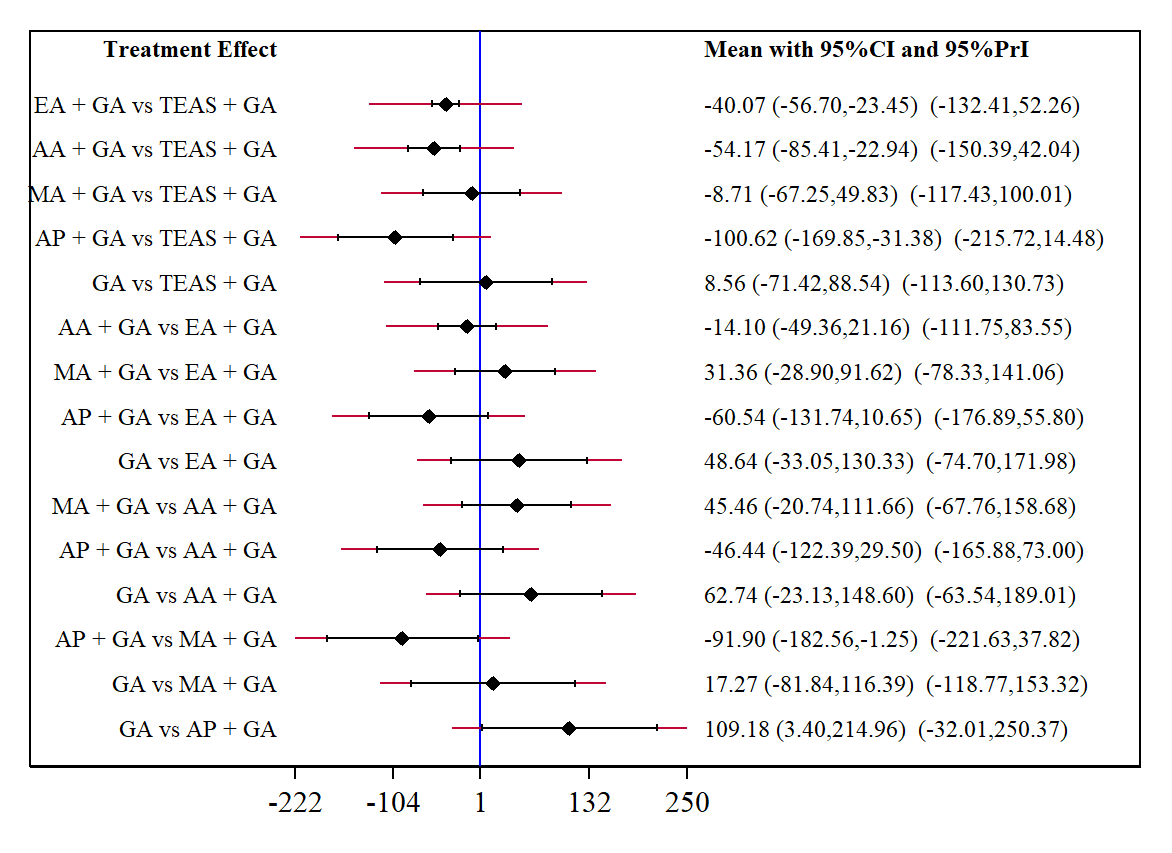


**Figure S3-1. Predictive intervals plot for total dose of** **propofol**

Note: The graph presents the network estimates for all pairwise comparisons. Black horizontal lines represent the credible intervals, and vertical lines represent the predictive intervals. TEAS: transcutaneous electrical acupoint stimulation; MA: manual acupuncture; EA: electroacupuncture; AA: auricular acupuncture; AP: acupressure; GA: general anesthesia


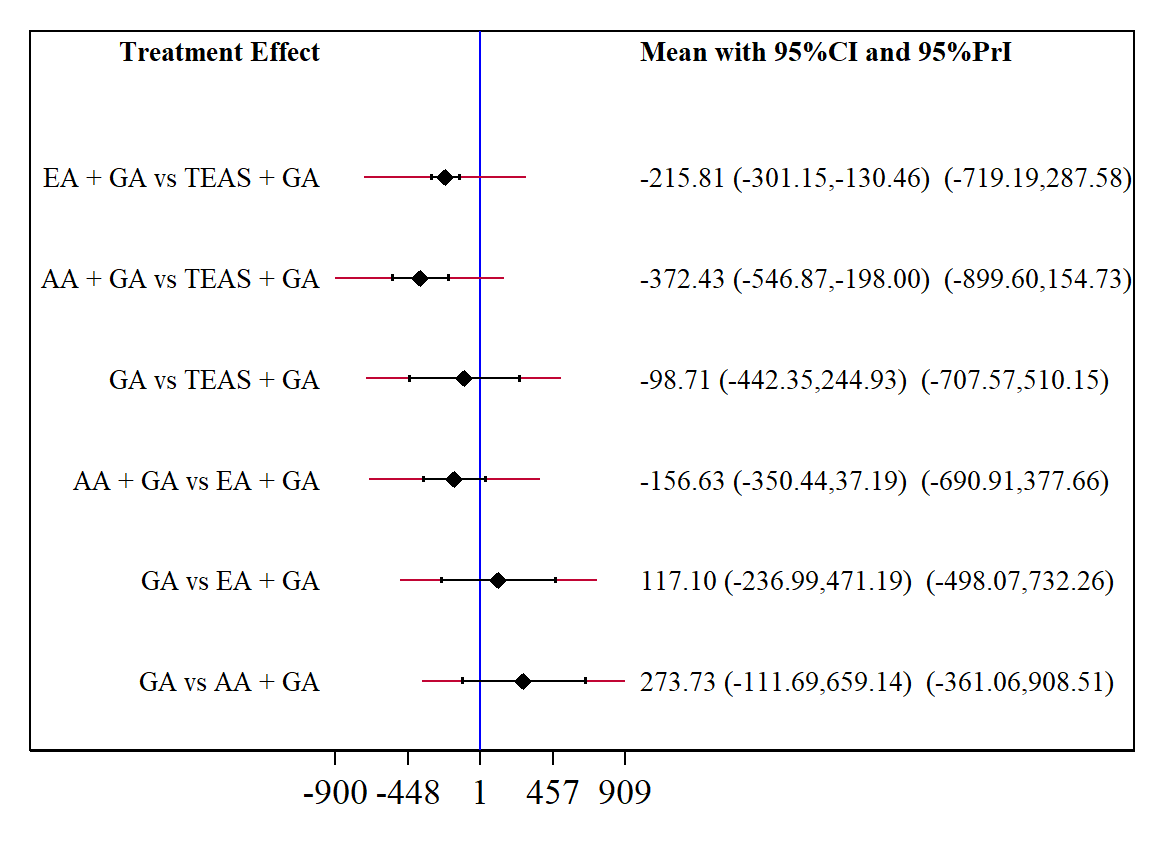


**Figure S3-2. Predictive intervals plot for total dose of remifentanil** Note: The graph presents the network estimates for all pairwise comparisons. Black horizontal lines represent the credible intervals, and vertical lines represent the predictive intervals. TEAS: transcutaneous electrical acupoint stimulation; EA: electroacupuncture; AA: auricular acupuncture; GA: general anesthesia

## Appendix 10

## Comparison-adjusted funnel plots


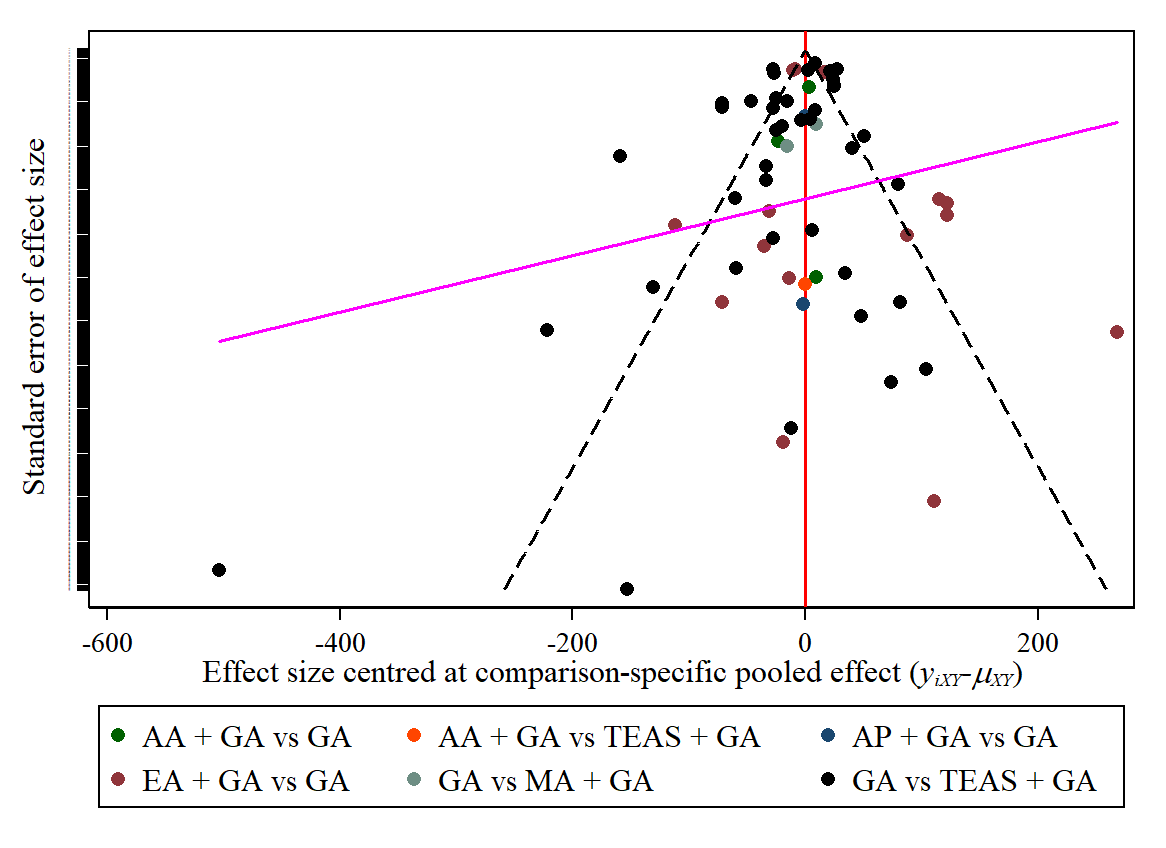


**Figure S4-1. Comparison-adjusted funnel plot for propofol**

Note: TEAS: transcutaneous electrical acupoint stimulation; MA: manual acupuncture; EA: electroacupuncture; AA: auricular acupuncture; AP: acupressure; GA: general anesthesia


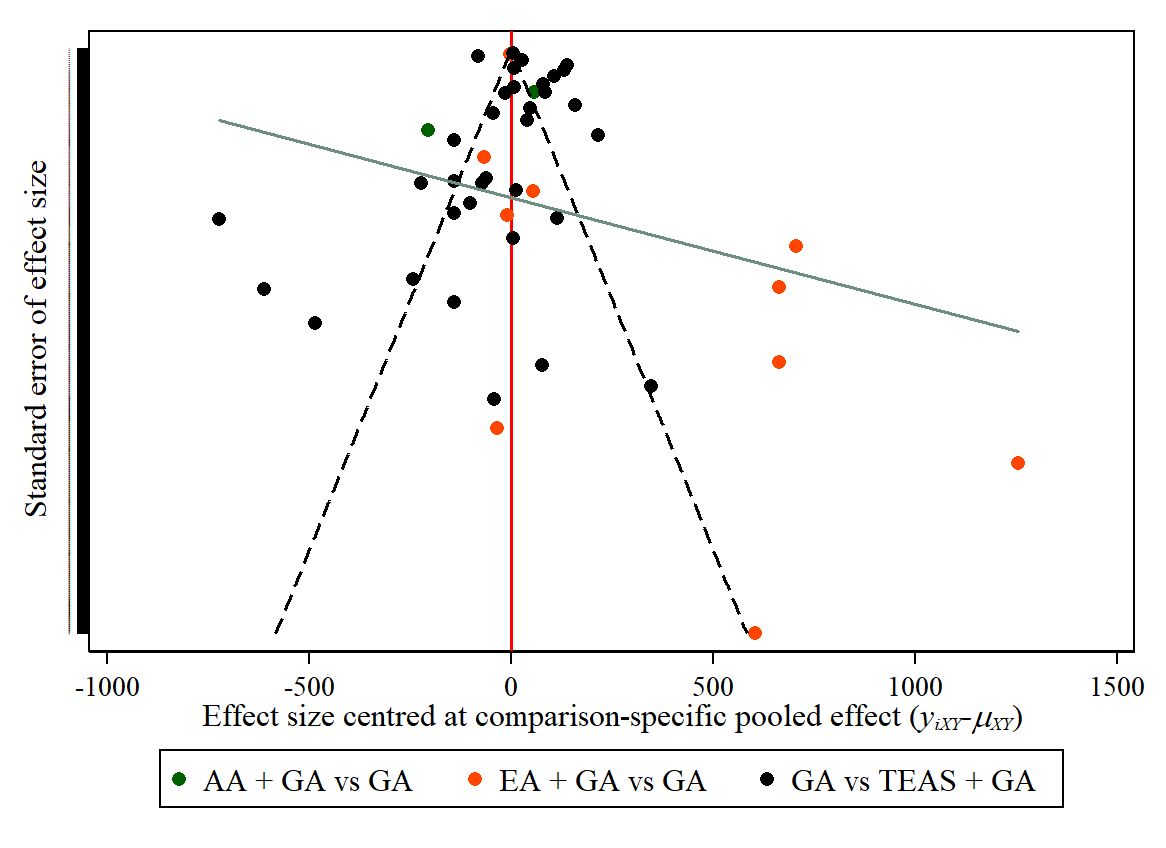


**Figure S4-2. Comparison-adjusted funnel plot for remifentanil**

Note: TEAS: transcutaneous electrical acupoint stimulation; EA: electroacupuncture; AA: auricular acupuncture; GA: general anesthesia

## Appendix 11

## Contribution summary of risk of bias assessments

**Table S5a-1. Contribution summary of risk of bias assessments for any direct comparisons included in the network meta-analysis on propofol**

| **Direct comparison** | **Risk of bias assessment** |
| --- | --- |
| TEAS + GA vs. AA + GA | Moderate |
| TEAS + GA vs. GA | Moderate |
| EA + GA vs. GA | Moderate |
| AA + GA vs. GA | Moderate |
| MA + GA vs. GA | Moderate |
| AP + GA vs. GA | Moderate |

Note: TEAS: transcutaneous electrical acupoint stimulation; MA: manual acupuncture; EA: electroacupuncture; AA: auricular acupuncture; AP: acupressure; GA: general anesthesia

**Table S5a-2. Contribution summary of risk of bias assessments for any direct comparisons included in the network meta-analysis on remifentanil**

| **Direct comparison** | **Risk of bias assessment** |
| --- | --- |
| TEAS + GA vs. GA | Moderate |
| EA + GA vs. GA | Moderate |
| AA + GA vs. GA | Moderate |

Note: TEAS: transcutaneous electrical acupoint stimulation; EA: electroacupuncture; AA: auricular acupuncture; GA: general anesthesia

**Table S5b-1. The contribution of direct comparisons to mixed or indirect comparisons by risk of bias classification on propofol**

| **Comparisons** | **Risk of bias assessment** | | |
| --- | --- | --- | --- |
|  | **Low (%)** | **Moderate (%)** | **High (%)** |
| TEAS + GA vs. AA + GA | 0 | 100 | 0 |
| TEAS + GA vs. GA | 0 | 100 | 0 |
| EA + GA vs. GA | 0 | 100 | 0 |
| AA + GA vs. GA | 0 | 100 | 0 |
| MA + GA vs. GA | 0 | 100 | 0 |
| AP + GA vs. GA | 0 | 100 | 0 |

Note: TEAS: transcutaneous electrical acupoint stimulation; MA: manual acupuncture; EA: electroacupuncture; AA: auricular acupuncture; AP: acupressure; GA: general anesthesia

**Table S5b-2. The contribution of direct comparisons to mixed or indirect comparisons by risk of bias classification on remifentanil**

| **Comparisons** | **Risk of bias assessment** | | |
| --- | --- | --- | --- |
|  | **Low (%)** | **Moderate (%)** | **High (%)** |
| TEAS + GA vs. GA | 0 | 100 | 0 |
| EA + GA vs. GA | 0 | 100 | 0 |
| AA + GA vs. GA | 0 | 100 | 0 |

Note: TEAS: transcutaneous electrical acupoint stimulation; EA: electroacupuncture; AA: auricular acupuncture; GA: general anesthesia

## Appendix 12

## Evaluation of the quality of evidence using GRADE framework

**Table S6-1. Evaluation of the quality of evidence using GRADE framework for propofol**

| **Comparison** | **Study limitation** | **Imprecision** | **Heterogeneity and inconsistency** | **Indirectness** | **Publication bias** | **Confidence in WMD for propofol** |
| --- | --- | --- | --- | --- | --- | --- |
| TEAS + GA vs. GA | 100% of the estimate from studies at moderate risk. | MD = -39.99,  95% CI: -57.96 to -22.73 | Moderate heterogeneity according to I^2^ (87.3%) and *P*-value (< 0.001) in direct comparisons. | The treatment effect was significantly influenced by clinical modifiers (e.g.; mean age, mean surgery time) in the subgroup analyses. | The funnel plot for the direct comparison is not suggestive of any dominant publication bias. | Moderate (Downgrade by one level due to and indirectness) |
| EA + GA vs. GA | 100% of the estimate from studies at moderate risk. | MD = -54.25,  95% CI: -87.25 to -22.37 | Moderate heterogeneity according to I^2^ (81.4%) and *P*-value (< 0.001) in direct comparisons. | The treatment effect was significantly influenced by clinical modifiers (e.g.; mean age, mean surgery time) in the subgroup analyses. | The funnel plot for the direct comparison is not suggestive of any dominant publication bias. | Moderate (Downgrade by one level due to indirectness) |
| AA + GA vs. GA | 100% of the estimate from studies at moderate risk. | MD = -9.36,  95% CI: -72.26 to -51.97 | Mild heterogeneity according to I^2^ (0%) and  *P*-value (0.531) in direct comparisons. | The treatment effects were not significantly influenced by clinical modifiers in the subgroup analyses. | Undetectable by routine method. | Moderate (Downgrade by one level due to imprecision) |
| MA + GA vs. GA | 100% of the estimate from studies at moderate risk. | MD = -101.26,  95% CI: -172.98 to -27.06 | Mild heterogeneity according to I^2^ (0%) and  *P*-value (0.417) in direct comparisons. | The treatment effect was significantly influenced by clinical modifiers (e.g.; mean surgery time) in the subgroup analyses. | Undetectable by routine method. | Moderate (Downgrade by one level due to indirectness) |
| AP + GA vs. GA | 100% of the estimate from studies at moderate risk. | MD = 8.37,  95% CI: -75.33 to 91.52 | Mild heterogeneity according to I^2^ (0%) and  *P*-value (0.975) in direct comparisons. | The treatment effects were not significantly influenced by clinical modifiers in the subgroup analyses. | Undetectable by routine method. | Moderate (Downgrade by one level due to imprecision) |
| TEAS + GA vs. AA + GA | 100% of the estimate from studies at moderate risk. | MD = -30.62,  95% CI: -94.36 to 32.47 | There was only one head-to-head study and no heterogeneity.  No inconsistency between direct and indirect estimate (Node-split *P* = 0.165). | The treatment effects were not significantly influenced by clinical modifiers in the subgroup analyses. | Undetectable by routine method. | Moderate (Downgrade by one level due to imprecision) |
| Ranking of treatment | 100% of the estimate from studies at moderate risk. | SUCRA plots suggested precision in a ranking of treatments. | Severe heterogeneity in network meta-analyses according to global I^2^ (89.43%).  No significant inconsistency in test of global inconsistency (*P* = 0.296), and no local inconsistency. | The treatment effects were not significantly influenced by clinical modifiers in the subgroup analyses. | The comparison-adjusted funnel plot for the network is not suggestive of any dominant publication bias. | Moderate (Downgrade by two levels due to heterogeneity) |

Note: TEAS: transcutaneous electrical acupoint stimulation; MA: manual acupuncture; EA: electroacupuncture; AA: auricular acupuncture; AP: acupressure; GA: general anesthesia

**Table S6-2. Evaluation of the quality of evidence using GRADE framework for remifentanil**

| **Comparison** | **Study limitation** | **Imprecision** | **Heterogeneity and inconsistency** | **Indirectness** | **Publication bias** | **Confidence in WMD for remifentanil** |
| --- | --- | --- | --- | --- | --- | --- |
| TEAS + GA vs. GA | 100% of the estimate from studies at moderate risk. | MD = -215.77,  95% CI: -305.23 to -128.04) | Severe heterogeneity according to I^2^ (97.6%) and *P*-value (< 0.001) in direct comparisons. | The treatment effect was significantly influenced by clinical modifiers (e.g.; mean age, mean surgery time) in the subgroup analyses. | The funnel plot for the direct comparison is not suggestive of any dominant publication bias. | Low (Downgrade by two levels due to heterogeneity and indirectness) |
| EA + GA vs. GA | 100% of the estimate from studies at moderate risk. | MD = -372.33,  95% CI: -558.44 to -196.43 | Severe heterogeneity according to I^2^ (93.5%) and *P*-value (< 0.001) in direct comparisons. | The treatment effect was significantly influenced by clinical modifiers (e.g.; mean age, mean surgery time) in the subgroup analyses. | The funnel plot for the direct comparison is not suggestive of any dominant publication bias. | Low (Downgrade by two levels due to heterogeneity and indirectness) |
| AA + GA vs. GA | 100% of the estimate from studies at moderate risk. | MD = -99.79,  95% CI: -457.6 to 268.35 | Severe heterogeneity according to I^2^ (96.9%) and *P*-value (< 0.001) in direct comparisons. | The treatment effects were not significantly influenced by clinical modifiers in the subgroup analyses. | Undetectable by routine method. | Low (Downgrade by one level due to heterogeneity and imprecision) |
| Ranking of treatment | 100% of the estimate from studies at moderate risk. | SUCRA plots suggested precision in a ranking of treatments. | Severe heterogeneity in network meta-analyses according to global I^2^ (99.78%). | The treatment effects were not significantly influenced by clinical modifiers in the subgroup analyses. | The comparison-adjusted funnel plot for the network is not suggestive of any dominant publication bias. | Moderate (Downgrade by two levels due to heterogeneity) |

Note: TEAS: transcutaneous electrical acupoint stimulation; EA: electroacupuncture; AA: auricular acupuncture; GA: general anesthesia

## Appendix 13

**Sensitivity network meta-analyses for propofol and remifentanil compared with general anesthesia**

| **TEAS + GA** | **-272 (-521.53, -26.7)** | 36.76 (-495.43, 577.21) | NA | **266.09 (163.89, 370.81)** |
| --- | --- | --- | --- | --- |
| 10.73 (-31.72, 55.79) | **EA + GA** | 306.4 (-253.58, 885.02) | NA | **537.75 (315.93, 765.24)** |
| -50.64 (-161.07, 57.54) | -62.03 (-176.11, 50.37) | **AA + GA** | NA | 230.75 (-304.13, 751.59) |
| 43.68 (-39.78, 126.52) | 32.89 (-56.06, 119.66) | 93.84 (-38.13, 228.04) | **MA + GA** | NA |
| **-56.63 (-80.8, -34.31)** | **-67.55 (-106.35, -31.29)** | -5.84 (-113.78, 101.02) | **-100.43 (-181.66, -21.44)** | **GA** |

**Figure S7. WMD (weighted mean difference) with 95%CI of network meta-analysis for propofol (lower triangle, No. of studies = 45) and remifentanil (upper triangle, No. of studies = 35)**

Note: Treatments were reported in alphabetical order. Results of network meta-analysis were listed in the lower triangle, and the estimation was calculated as the column-defining treatment compared with the row-defining treatment. NA: not available. TEAS: transcutaneous electrical acupoint stimulation; MA, manual acupuncture; EA, electroacupuncture; AA, auricular acupuncture; GA, general anesthesia.

## Appendix 14

**Subgroup network meta-analyses for propofol and remifentanil compared with general anesthesia**

**Table S8-1. Subgroup network meta-analyses for propofol compared with general anesthesia**

| **Characteristics** | **TEAS** | **Electroacupuncture** | **Auricular acupuncture** | **Manual acupuncture** | **Acupressure** |
| --- | --- | --- | --- | --- | --- |
| **All trials** | **-40 (-58, -23)** | **-54 (-87, -22)** | -9 (-72, 52) | **-101 (-173, -27)** | 8 (-75, 92) |
| **Mean age** |  |  |  |  |  |
| **< 60 years** | **-43 (-67, -18)** | **-63 (-110, -21)** | -14 (-110, 80) | NA | 8.8 (-120, 140) |
| **≥ 60 years** | -26 (-65, 13) | -31 (-88, 26) | -6.1 (-87, 78) | **-100 (-160, -39)** | 7.7 (-140, 150) |
| **Type of anesthesia** |  |  |  |  |  |
| **TIVA** | **-48 (-72, -24)** | **-56 (-97, -16)** | -12 (-84, 59) | **-100 (-190, -16)** | NA |
| **CIIA** | -21 (-47, 0.94) | -38 (-100, 13) | NA | NA | 8.4 (-57, 73) |
| **Compared with sham acupuncture + GA or GA** |  |  |  |  |  |
| **Sham acupuncture + GA** | **-23 (-40, -8.5)** | -14 (-140, 110) | 4.4 (-38, 49) | NA | 8.3 (-47, 63) |
| **GA** | **-57 (-90, -25)** | **-59 (-100, -20)** | -67 (-210, 76) | **-100 (-190, -8.4)** | NA |
| **Number of acupoints** |  |  |  |  |  |
| **≤ 3** | **-39 (-58, -20)** | **-45 (-81, -10)** | 21 (-63, 110) | **-100 (-160, -41)** | 8.2 (-61, 78) |
| **> 3** | **-46 (-89, -7.9)** | -71 (-150, 1.6) | -32 (-150, 84) | NA | NA |
| **Administration** |  |  |  |  |  |
| **Pre-surgery** | **-41 (-75, -10)** | **-62 (-110, -62)** | 20 (-100, 140) | **-100 (-180, -17)** | 7.6 (-110, 130) |
| **Pre-surgery + during surgery** | **-41 (-64, -18)** | -42 (-99, 10) | -24 (-110, 52) | NA | 10 (-150, 170) |
| **Type of surgery** |  |  |  |  |  |
| **Abdominal surgery** | **-45 (-85, -12)** | **-99 (-160, -42)** | 5.7 (-76, 87) | **-100 (-180, -17)** | 7.5 (-110, 120) |
| **Breast surgery** | -39 (-70, 0.33) | NA | -57 (-180, 70) | NA | NA |
| **Orthopedic surgery** | **-33 (-64, -3.7)** | -21 (-59, 26) | NA | NA | NA |
| **Neck surgery** | -62 (-210, 92) | NA | NA | NA | NA |
| **Thoracic surgery** | -38 (-190, 110) | -54 (-180, 58) | NA | NA | 8.8 (-260, 270) |
| **Surgery duration** |  |  |  |  |  |
| **< 120 min** | **-31 (-60, -3.5)** | **-97 (-150, -40)** | -9 (-95, 71) | -89 (-190, 18) | 7.7 (-100, 110) |
| **≥ 120 min** | -33 (-73, 0.61) | -7.7 (-68, 51) | -5.7(-120, 110) | NA | 9.9 (-160, 180) |

Note: CIIA: Combined intravenous and inhalation anesthesia; TIVA: total intravenous anesthesia; NA: not available. TEAS: transcutaneous electrical acupoint stimulation

**Table S8-2. Subgroup network meta-analyses for** **remifentanil compared with general anesthesia**

| **Characteristics** | **TEAS** | **Electroacupuncture** | **Auricular acupuncture** |
| --- | --- | --- | --- |
| **All trials** | **-216 (-305, -128)** | **-372 (-558, -196)** | 100 (-458, 268) |
| **Mean age** |  |  |  |
| **< 60 years** | **-180 (-270, -85)** | **-410 (-590, -240)** | 32 (-400, 480) |
| **≥ 60 years** | -82 (-180, 10) | -45 (-260, 180) | **-230 (-450, -4.3)** |
| **Type of anesthesia** |  |  |  |
| **TIVA** | **-220 (-330, -110)** | **-410 (-620, -220)** | -96 (-480, 290) |
| **CIIA** | **-200 (-390, -19)** | -100 (-700, 490) | NA |
| **Compared with sham acupuncture + GA or GA** |  |  |  |
| **Sham acupuncture + GA** | **-200 (-280, -120)** | -68 (-380, 240) | -100 (-360, 160) |
| **GA** | **-240 (-490, -7)** | **-480 (-780, -210)** | NA |
| **Number of acupoints** |  |  |  |
| **≤ 3** | **-210 (-320, -110)** | **-280 (-480, -76)** | 36 (-440, 520) |
| **> 3** | **-220 (-410, -32)** | **-600 (-1000, -200)** | -230 (-890, 460) |
| **Administration** |  |  |  |
| **Pre-surgery** | -170 (-350, 8) | **-380 (-680, -93)** | 33 (-560, 630) |
| **Pre-surgery + during surgery** | **-240 (-360, -130)** | **-370 (-640, -120)** | -230 (-750, 290) |
| **Type of surgery** |  |  |  |
| **Abdominal surgery** | **-190 (-340, -35)** | -140 (-500, 190) | -99 (-470, 280) |
| **Breast surgery** | **-280 (-370, -190)** | NA | NA |
| **Orthopedic surgery** | -220 (-640, 220) | -270 (-840, 290) | NA |
| **Neck surgery** | **-170 (-290, -66)** | NA | NA |
| **Thoracic surgery** | -310 (-1000, 350) | **-680 (-1300, -75)** | NA |
| **Surgery duration** |  |  |  |
| **< 120 min** | **-140 (-210, -84)** | 21 (-240, 280) | 34 (-220, 290) |
| **≥ 120 min** | -140 (-300, 27) | **-400 (-620, -190)** | -230 (-720, 260) |

Note: CIIA: Combined intravenous and inhalation anesthesia; TIVA: total intravenous anesthesia; NA: not available. TEAS: transcutaneous electrical acupoint stimulation
